# TMEM203 is a binding partner and regulator of STING-mediated inflammatory signaling in macrophages

**DOI:** 10.1073/pnas.1901090116

**Published:** 2019-07-25

**Authors:** Yang Li, Sharmy J. James, David H. Wyllie, Claire Wynne, Agnes Czibula, Ahmed Bukhari, Katherine Pye, Seri Musfirah Bte Mustafah, Roberta Fajka-Boja, Eniko Szabo, Adrienn Angyal, Zoltan Hegedus, Laszlo Kovacs, Adrian V. S. Hill, Caroline A. Jefferies, Heather L. Wilson, Zhang Yongliang, Endre Kiss-Toth

**Affiliations:** ^a^Department of Infection, Immunity and Cardiovascular Disease, University of Sheffield, S10 2RX Sheffield, United Kingdom;; ^b^Department of Microbiology and Immunology, Yong Loo Lin School of Medicine, National University of Singapore, Singapore 117545;; ^c^Immunology Programme, The Life Science Institute, National University of Singapore, Singapore 117597;; ^d^Jenner Institute, Oxford University, OX3 9DU Oxford, United Kingdom;; ^e^School of Biological and Health Sciences, Technological University Dublin, Dublin 8, Ireland;; ^f^Department of Genetics, Biological Research Centre of the Hungarian Academy of Sciences, Szeged, H-6726, Hungary;; ^g^Department of Biochemistry and Medical Chemistry, University of Pécs, Pécs 7624, Hungary;; ^h^Department of Rheumatology and Immunology, Faculty of Medicine, Albert Szent-Györgyi Health Centre, University of Szeged, H-6725 Szeged, Hungary;; ^i^Department of Biomedical Sciences and Rheumatology, Cedars-Sinai Medical Center, Los Angeles, CA 90048

**Keywords:** TMEM203, STING, lupus, interferon signaling, STIM1

## Abstract

Regulation of IFN signaling is critical in host recognition and response to pathogens while its dysregulation underlies the pathogenesis of several chronic diseases. STimulator of IFN Genes (STING) has been identified as a critical mediator of IFN inducing innate immune pathways, but little is known about direct coregulators of this protein. We report here that TMEM203, a conserved putative transmembrane protein, is an intracellular regulator of STING-mediated signaling. We show that TMEM203 interacts, functionally cooperates, and comigrates with STING following cell stimulation, which in turn leads to the activation of the kinase TBK1, and the IRF3 transcription factor. This induces target genes in macrophages, including IFN-β. Using *Tmem203* knockout bone marrow-derived macrophages and transient knockdown of TMEM203 in human monocyte-derived macrophages, we show that TMEM203 protein is required for cGAMP-induced STING activation. Unlike *STING*, *TMEM203* mRNA levels are elevated in T cells from patients with systemic lupus erythematosus, a disease characterized by the overexpression of type I interferons. Moreover, *TMEM203* mRNA levels are associated with disease activity, as assessed by serum levels of the complement protein C3. Identification of TMEM203 sheds light into the control of STING-mediated innate immune responses, providing a potential novel mechanism for therapeutic interventions in STING-associated inflammatory diseases.

## Significance

Activators of interferons have received a great deal of interest in recent decades, both due to the central role they play in host defense against a range of pathogens, as well as the now well-recognized importance of dysregulated interferon activation/signaling in the pathogenesis of a number of highly prevalent and hard-to-treat diseases, such as systemic lupus erythematosus (SLE). Therefore, novel regulators of interferon activation are being sought as they may provide better targets to treat these diseases. We report the discovery of TMEM203 as an SLE-associated gene and a regulator of ligand-dependent activation of interferon production via STING. Thus, our work could form the basis of a novel therapeutic strategy for the treatment of interferonopathies, including SLE.

Innate immune sensing of microbial infections involves pathogen pattern recognition receptors (PRRs), such as Toll-like receptors (TLRs). Many TLR-dependent and -independent innate signaling systems, including NOD-like receptors and systems recognizing intracellular DNA (1, 2), activate the TBK1/IRF3 axis, a pathway of fundamental importance in immune defense in both bacterial and viral diseases (3). Activation of this pathway, which is of great phylogenetic antiquity (4), results in the production of IFN-β, a cytokine critical for host defense against both viruses and bacteria. As increasing evidence links the PRR/TBK1/IRF3 axis to autoimmune disease (including systemic lupus erythematosus [SLE]) (5), vaccine responses (6), and the development of malignancy (7), the identification of regulators of this pathway may reveal novel therapeutic targets.

One important component mediating activation of the TBK1/IRF3 pathway is the endosomal multitransmembrane protein STimulator of IFN Genes (STING) (2, 8). STING is activated by the double-stranded DNA (dsDNA) sensor IFI16, or by direct binding to bacteria-secreted cyclic dinucleotide c-di-AMP, c-di-GMP, and 3′3′-cGAMP, as well as cGAS-catalyzed (9, 10) mammalian ligand 2′3′-cGAMP. Its critical role is proven both by the lack of IFN induction following viral, bacterial, or synthetic DNA stimulation in STING-deficient cells (2, 11), and by the increased sensitivity of STING-deficient mice to DNA viruses such as HSV-1 (2). Constitutively activated STING variants have been found in patients diagnosed with severe symptoms of type I interferonopathy, leading to diseases such as STING-associated vasculopathy with onset in infancy (SAVI) (12), systemic lupus erythematosus (SLE) (5), and familial chilblain lupus (FCL) (13). The importance of STING activity in health and disease has also been the subject of recent reviews (14).

Following STING activation, the serine/threonine kinase TBK1 is recruited to the cytosolic face of the endo-lysosome/endoplasmic reticulum (ER) (15). At these intracellular vesicles, STING is targeted for a regulatory (K27-linked) ubiquitination by the E3 ubiquitin ligase autocrine motility factor receptor (AMFR), triggering its activation and the subsequent phosphorylation of the transcription factor IRF3 (16). Once phosphorylated, IRF3 dimerizes and translocates to the nucleus where it drives the expression of genes containing IRF binding sites in their promoter, predominantly the type I interferons IFN-α and IFN-β (15). Postactivation, STING is sorted to the endo-lysosomes where it is targeted by microtubule-associated proteins 1A/1B light chain 3B (LC3) and autophagy-related protein 9a (Atg9a) to attenuate its functions (17, 18). Recent work identified the Ca^2+^ sensor STIM1 as a critical STING binding partner, responsible for its retention in the ER, thus preventing spontaneous activation (19). However, little is known about the mechanisms and interacting proteins that drive STING translocation to the endo-lysosomal compartment.

While most studies have described STING as a critical component in cytosolic nucleic acid recognition, STING has also been shown to play a role in augmented IRF3 activation and type I IFN (IFN-I) induction upon concomitant ER stress and LPS stimulation (2) via late-TLR4 signaling (20). Despite the fundamental importance of STING in both antibacterial and antiviral immunity, its partners remain largely unknown, with many aspects of its mechanism of action still being poorly understood.

In previous functional screens discovering novel regulators of inflammation (21), we reported the identification of TMEM203 as a previously unknown proinflammatory gene in mouse macrophages (21). Here, we demonstrate that TMEM203, a protein that was recently shown to be endosomal and interacts with the pleiotropic inositol phosphate signaling pathway protein IP3R (22), is associated with SLE disease activity, forms a functional and ligand-dependent complex with STING, and promotes its translocation to endo-lysosomes. TMEM203 is a regulator of signaling pathways activated in response to diverse bacterial and viral stimuli, including cyclic dinucleotides, and can serve as a future therapeutic target to attenuate STING-mediated pathological IFN activation.

## Results

### TMEM203 Is an Evolutionarily Conserved Putative Transmembrane Protein, Regulated by Inflammatory Stimuli.

We have previously identified multiple regulators of inflammatory signaling in macrophages by genome-wide expression screening for genes which drove expression of the inflammatory chemokine *Cxcl2* when transfected into RAW 264.7 cells (21); TMEM203 was one such protein. Multiple alignment of TMEM203 orthologs from a wide range of species demonstrated that TMEM203 is an evolutionally highly conserved gene (*SI Appendix*, Fig. S1), encoding a 136-amino acid protein, with the mouse and human orthologs being 98% identical. Interestingly, a survey of the GenBank database revealed that only a single copy of this gene is present in both invertebrate and vertebrate species; TMPred (23) predicts four putative membrane-spanning helices (*SI Appendix*, Fig. S1).

**Fig. 1.**
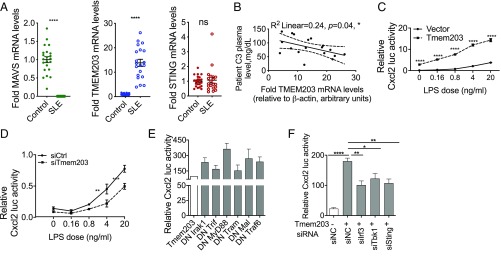
(*A* and *B*) TMEM203 mRNA level is elevated in a cohort of SLE patients. (*A*) MAVS, TMEM203, and STING mRNA levels in PHA-L–activated T cells were assessed by RT-qPCR from a cohort of treatment-naive systemic erythematous lupus (SLE) patients and in healthy individuals. (*B*) Spearman correlation coefficient of C3 complement plasma level was assessed against fold TMEM203 mRNA induction in SLE patients (95% CI: dashed lines). (*C*) Overexpression of Tmem203 augments LPS-induced Cxcl2 activation. RAW 264.7 cells were transfected with the *Cxcl2*-pLuc and *EF1*-rLuc reporters, and with *Tmem203* expression plasmid (dashed line) or empty control vector (solid line). Cells were stimulated with the stated concentration of LPS for 6 h. Two-way ANOVA with Sidak correction, *n* = 3. (*D*) Knockdown of *Tmem203* impairs LPS-induced *Cxcl2* activation. RAW 264.7 cells were transfected with the *Cxcl2*-pLuc and *EF1*-rLuc reporters, and with siRNA against *Tmem203* (dashed line) or nontargeting si-scrambled (solid line). Cells were stimulated with the stated concentration of LPS for 6 h. Two-way ANOVA with Sidak correction, *n* = 3. (*E*) Dominant negative (DN) signaling molecules fail to inhibit *Tmem203* induced *Cxcl2* promoter activity. RAW 264.7 cells were transfected as in *F*, and the ability of the DN constructs to block overexpressed *Tmem203*-induced *Cxcl2* activation was tested, *n* = 2. (*F*) Tmem203 induces Cxcl2 promoter via the Sting/Tbk1/Irf3 pathway. RAW 264.7 cells were transfected with the *Cxcl2*-pLuc, *TK*-rLuc reporters, and Tmem203 expression plasmid, together with siRNA as indicated. One-way ANOVA, *n* = 3. PHA-L: Phytohaemagglutinin-L. *P < 0.05, **P < 0.01, ****P < 0.0001; ns: not significant.

Dysregulated expression of key innate immune signaling molecules has previously been linked to the development of human pathologies, including systemic lupus erythematous (SLE), an inflammatory disease often characterized by both the recruitment of immune cells, including T lymphocytes, and their excessive type I IFN production in the affected tissues. Thus, we have analyzed the mRNA levels of two well-characterized intracellular signaling regulators, mitochondrial antiviral signaling protein (MAVS) and STING, as well as TMEM203, in T cells isolated from the blood of recently diagnosed, treatment-naive SLE patients. Both MAVS and STING have previously been implicated in driving IFN production in SLE (24, 25). We found significantly reduced, almost abolished, MAVS expression, with marked up-regulation of TMEM203 (mean 13.8 ± SD 6.5-fold induction, lower:upper interquartile = 8.6:18.8) (Fig. 1*A*). Further, TMEM203 mRNA levels in T cells from treatment-naive SLE patients inversely correlated with the plasma levels of complement factor C3 (Fig. 1*B*), a clinically used marker of activated innate immunity. A pilot analysis of MAVS protein levels in T cells isolated from SLE versus healthy control subjects has also shown a trend toward reduced MAVS expression in SLE (*SI Appendix*, Fig. S2*A*). Taken together, our data suggest that TMEM203 may play a role in the development of this disease and that this activity might be coupled to excessive production of type I IFN.

A search in the transcriptome database thebiogps.org (26) indicated that TMEM203 is highly expressed in myeloid cells, including macrophages, in addition to T cells. Since our expression screen showed that TMEM203 may act as a signaling regulator in myeloid cells (21), we first tested how chemokine expression in murine macrophages is controlled by TMEM203. Compared with controls, *Cxcl2* promoter activity in LPS-stimulated RAW 264.7 cells was enhanced by the overexpression of *Tmem203* (Fig. 1*C*), while being impaired by the siRNA-mediated down-regulation of *Tmem203* (Fig. 1*D*), suggesting that TMEM203 is likely to act downstream of Toll-like receptors (TLRs) and/or other innate immune sensors.

We next characterized the molecular pathways for *Tmem203* action, using *Cxcl2* promoter activity as a surrogate of the activation of canonical TLR pathways (27). While LPS-induced activation of a *Cxcl2*-luciferase reporter in RAW 264.7 cells was blocked (*SI Appendix*, Fig. S2*B*), the *Tmem203*-induced *Cxcl2* activation (∼3-fold above baseline) was not inhibited by the expression of the dominant negative forms of signaling mediators of canonical LPS signaling: *Irak1*, *Trif*, *MyD88*, *Tram*, *Mal*, and *Traf6* (Fig. 1*E*). Similarly, pharmacological inhibitors of mitogen-activated protein kinase (MAPK) impaired LPS-, but not overexpressed *Tmem203*-induced, *Cxcl2* promoter activities (*SI Appendix*, Fig. S2*C*). Therefore, we concluded that, while *TMEM203* is a proinflammatory mediator/effector, it is likely to act independently from the canonical TLR and MAPK networks. We therefore explored whether TMEM203 acts on the noncanonical inflammatory pathway since LPS also induces the TRIF-TRAM/TBK1/IRF3 signaling axis via endosomal “late signaling,” leading to activation of multiple inflammatory cytokines, including interferons (28). The TBK1/IRF3 axis is known to couple to the protein STING, a critical regulator of cytosolic double-stranded DNA detection (29, 30), and whose activities are mostly independent of MAPK and canonical TLR mediators (15, 30, 31). Activation of STING induces TBK1 phosphorylation (15), which subsequently induces IRF3 or NF-κB to elicit the type I IFN response and a variety of proinflammatory cytokines, including TNF, IL-6, and several chemokines (15).

Since Shambharkar et al. (22) have previously reported that TMEM203 is localized on ER membranes and that our above data suggested that TMEM203 is closely related to chemokine expression and SLE disease indications, we hypothesized that TMEM203 may regulate STING-mediated signaling events. Therefore, a *Tmem203* overexpressing plasmid construct and siRNA against the STING-signaling effectors *Tbk1* and *Irf3* were cotransfected into RAW 264.7 cells, followed by the measurement of *Cxcl2* promoter activities. As *Tmem203* overexpression resulted in elevated *Cxcl2* activity and the simultaneous suppression of *Sting* or its downstream regulators significantly reduced it (Fig. 1*F*), we concluded that TMEM203 potentially acts upstream or in parallel to a STING-dependent signaling pathway.

### TMEM203 Interacts with STING and Competes for STING Binding with STIM1.

**Fig. 2.**
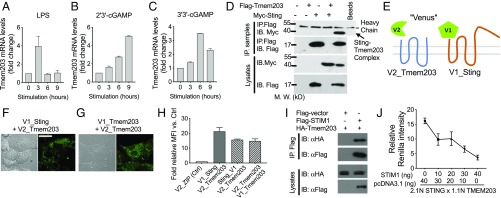
TMEM203 interacts with STING and STIM1. (*A*–*C*) *Tmem203* expression is transiently induced by inflammatory stimuli. Murine bone marrow-derived macrophages were stimulated with LPS (*A*), 2′3′-cGAMP (*B*), or 3′3′-cGAMP (*C*) for the time indicated. The expression of *Tmem203* was determined by RT-qPCR, *n* = 2. (*D*) TMEM203 coprecipitates with STING. HEK293 T cells were transfected with either empty vector, FLAG-TMEM203, or Myc-STING. TMEM203-containing complexes were immunoprecipitated (IP) using anti-FLAG–coated beads and blotted for Myc and FLAG, as indicated. Lysates were also immunoblotted for Myc and FLAG. *n* = 2. (*E*) Illustration of TMEM203-STING interaction by PCA. Tmem203 was tagged at its N terminus with the V1 fragment of Venus yellow fluorescent protein while Sting was tagged at its N terminus with the V2 Venus fragment. Both Tmem203 and Sting proteins are predicted to encode for four transmembrane domains (22, 54). Thus, this arrangement was predicted to localize the V1 and V2 tags to the same side of the lipid membrane. (*F* and *G*). TMEM203 and its complex with STING are located in the cytoplasm, in perinuclear structures. HeLa cells were transfected with the above-described Venus fusion protein expression plasmids. The “Venus” fluorescent signal, demonstrating TMEM203-STING interaction (*F*) or TMEM203 dimerization (*G*), was visualized. (Magnification, 80×.) (Scale bar: 20 µm.) (*H*) TMEM203 forms dimers and interacts with STING in live cells. HEK293 T cells were cotransfected with the indicated fusion protein expression vectors; the Venus fluorescence signal was detected by flow cytometry. *n* = 4. (*I*) TMEM203 coprecipitates with STIM1: HEK293 T cells were transfected with either empty vector, HA-TMEM203, or FLAG-STIM1. TMEM203-containing complexes were immunoprecipitated (IP) using anti-FLAG–coated beads and blotted for HA and FLAG, as indicated. Lysates were also immunoblotted for HA and FLAG. *n* = 2. (*J*) TMEM203 and STIM1 compete for binding to STING: TMEM203 and STING were tagged with the 1.1 and 2.1 fragment of Renilla luciferase, respectively, to test for a molecular interaction between these proteins in live cells. Tmem203 and Sting fusion protein expression vectors, together with an increasing dose of STIM1 expression vector, were transfected into HEK293 T cells. Relative luciferase activity was assessed 24 h posttransfection, *n* = 3. IB: immunoblotting; MFI: mean fluorescence intensity.

To establish whether TMEM203 coregulates STING, *Tmem203* expression levels were examined first in mouse bone marrow-derived macrophages (isolated from C57/BL6 mice) after stimulation with LPS, endogenous STING ligand 2′3′-cGAMP (29), or microbial secreted STING ligand 3′3′-cGAMP (32). Each tested stimulus rapidly induced Tmem203 mRNA levels, albeit with different kinetics (Fig. 2 *A*–*C*).

Based on our observation that suppression of STING expression impaired TMEM203-induced *Cxcl2* activation (Fig. 1*F*) and that STING and TMEM203 are both localized in intracellular membranes (22, 33), we questioned whether STING and TMEM203 directly interact and coregulate the activation of an inflammatory response. Coimmunoprecipitation of Myc-Sting and Flag-Tmem203 in HEK293T cells (Fig. 2*D*) confirmed that a molecular complex is formed between these two proteins. To further validate the association of TMEM203 and STING, we used the Yellow Fluorescence Protein (YFP) (Venus derivative) fragment complementation assay (PCA), which is based on expressing each putative binding partner in fusion with either the N-terminal (V1) or C-terminal (V2) portion of YFP (34, 35) (Fig. 2*E*). When the two test proteins interact, the YFP fluorophore self-assembles in a cyclization reaction which is essentially irreversible (36). This stable fluorescent signal can be detected by flow cytometry or fluorescence microscopy. We used this technique to ask whether TMEM203 and STING can interact intracellularly (Fig. 2*E*) and observed a punctate membrane/vesicular distribution of fluorescence labeling the TMEM203/STING complex (Fig. 2*F*). Interestingly, a similar distribution of TMEM203 dimer (or higher order multimer) was also observed (Fig. 2*G*). As TMEM203 and STING are each predicted to contain four transmembrane domains, the positioning of the V1 or V2 PCA tags enabled us to map the relative orientation of the C and N termini of these proteins. In agreement with the proposed schematic model in Fig. 2*E*, TMEM203 and STING with either N terminus and C terminus tags can lead to the formation of a fluorescent complex (Fig. 2*H*, second and third bar) while a strong signal was also seen by TMEM203 dimerization (Fig. 2*H*, fourth bar). A further insight into molecular mechanisms TMEM203/STING activity and their potential common binding partners was gained by demonstrating that the recently discovered STING-interacting protein STIM1 (19) also interacts with TMEM203 (Fig. 2*I*) and that STIM1 was able to disrupt the TMEM203/STING complex in a dose-dependent manner (Fig. 2*J*), suggesting that STIM1 and TMEM203 may compete for STING binding.

### TMEM203 Down-Regulation Impairs cGAMP-Induced STING-Mediated Type I IFN Expression.

**Fig. 3.**
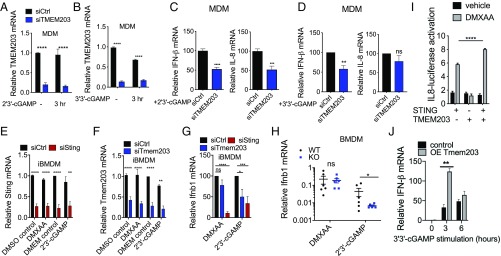
*TMEM203* down-regulation impairs cGAMP-induced STING-mediated type I IFN expression. (*A* and *B*) Efficient *TMEM203* knockdown in human monocyte-derived macrophages (MDMs). MDMs were transiently transfected by scrambled control (siCtrl) or TMEM203 targeting siRNA. Posttransfection, MDMs were left stimulated with ±4 µg/mL 2′3′-cGAMP (*A*) or 1 µg/mL 3′3′-cGAMP (*B*) for 3 h. *TMEM203* knockdown was quantified by RT-qPCR. Multiple Student’s *t* tests with Holm–Sidak corrections, *n* = 10 (*A*) and *n* = 4 (*B*). (*C* and *D*) *TMEM203* knockdown impairs 2′3′-cGAMP (*C*) and 3′3′-cGAMP (*D*) induced IFN-β production in MDMs. 2′3′-cGAMP (4 µg/mL) or 3′3′-cGAMP (1 µg/mL) stimulated (3 h) IFN-β production of siCtrl vs. siTMEM203-transfected MDMs from four donors was compared by normalizing *IFN-β* levels of siTMEM203-treated cells to the siCtrl treatment for each individual. Student’s *t* test, *n* = 4. (*E* and *F*) Efficient *Tmem203* (*E*) and *Sting* (*F*) knockdown in immortalized mouse bone marrow-derived macrophages (iBMDMs). iBMDMs were transiently transfected by scrambled control (siCtrl), Tmem203, or Sting targeting siRNA. Posttransfection, cells were left stimulated with ±25 µg/mL DMXAA or 20 µg/mL 2′3′-cGAMP for 3 h. Tmem203 and Sting knockdown was quantified by RT-qPCR. Multiple Student’s *t* tests with Holm–Sidak corrections, *n* = 4. (*G*) 2′3′-cGAMP, but not DMXAA, induced *Ifnb1* expression is impaired by *Tmem203* knockdown in iBMDMs. IFN-β induction by DMXAA (25 µg/mL) or 2′3′-cGAMP (20 µg/mL) stimulation (3 h) in the siTmem203 or siSting transfected iBMDMs was compared with that in the siCtrl-treated cells. One-way ANOVA, *n* = 4. (*H*) 2′3′-cGAMP, but not DMXAA, induced *Ifnb1* expression is impaired by *Tmem203* knockout in bone marrow-derived macrophages (BMDMs). BMDMs derived from WT or Tmem203 knockout C57BL/6 mice were stimulated with DMXAA (50 µg/mL) or 2′3′-cGAMP (10 µg/mL) for 3 h before *Ifnb1* expression levels were analyzed by RT-qPCR. Multiple Student’s *t* tests with Holm–Sidak corrections, *n* = 6. (*I*) *Tmem203* overexpression augments *Sting*-induced IL-8 activation. HEK293 T cells were cotransfected with *IFN-β* reporter and plasmids expressing Sting, Tmem203, or controls. Data are expressed as fold change in reporter induction relative to the control plasmid, with (gray bars) or without (black bars) stimulation of DMXAA (100 µg/mL, 6 h). Two-way ANOVA, *n* = 3. (*J*) *Tmem203* overexpression augments *Ifnb* activation in RAW 264.7 cells. Vector or Tmem203 overexpression (OE Tmem203) transfected RAW 264.7 cells were stimulated with 3′3′-cGAMP for the time indicated. The expression of *Ifnb* was determined by quantitative RT-qPCR. Student’s *t* tests, *n* = 3. DMEM: Dulbecco’s Modified Eagle Medium; DMSO: dimethyl sulfoxide. *P < 0.05, **P < 0.01, ***P < 0.001, ****P < 0.0001; ns: not significant.

STING predominantly mediates type I IFN activation in response to pathogen-associated cyclic-dinucleotide production in the cytoplasm (29, 30, 37). Building on these findings, we investigated the importance of TMEM203 in mediating STING activation in human monocyte-derived macrophages (MDMs). CD14-positive human monocytes (*SI Appendix*, Fig. S3*A*) were isolated from whole blood and differentiated into MDMs (*SI Appendix*, Fig. S3*B*) by macrophage colony-stimulating factor. MDMs were transfected with control or TMEM203 targeting siRNA, followed by a 3-h stimulation of these cells with 2′3′-cGAMP or 3′3′-cGAMP, 48 h posttransfection. RT-qPCR analysis of TMEM203 mRNA levels confirmed a highly robust (>70%) knockdown in siTMEM203-targeted MDMs (Fig. 3 *A* and *B*), which was accompanied by a significant inhibition of cGAMP-induced IFN-I expression in human MDM samples obtained from a cohort of healthy individuals (Fig. 3 *C* and *D*). While the amount of IFN-β mRNA produced in these samples showed donor-specific variation upon ligand stimulation (*SI Appendix*, Fig. S3 *C* and *D*), siTMEM203 treatment in each case reduced 2′3′-cGAMP and 3′3′-cGAMP induced IFN-β mRNA levels by ∼50% (Fig. 3 *C* and *D*). In contrast, levels of *IL-8* chemokine mRNA were not induced under both stimulation conditions (0- to 3-fold, depending on the specific donor or MDM isolation) although its levels were also reduced by *TMEM203* suppression in response to 2′3′-cGAMP treatment (Fig. 3*C*).

To expand on the above findings from primary human macrophages, we investigated whether TMEM203 displays similar STING regulatory behavior in mouse macrophages. siRNA knockdown of *Tmem203* or *Sting* was performed in immortalized bone marrow-derived macrophages (iBMDMs), followed by 3-h stimulation with the physiological STING ligand 2′3′-cGAMP or the synthetic ligand DMXAA, also known as Vadimesan (38), that selectively targets the mouse but not the human protein. Efficient suppression of both *Tmem203* and *Sting* was confirmed by RT-qPCR analysis (Fig. 3 *E* and *F*). Similar to MDMs, 2′3′-cGAMP robustly induced IFN-I expression in iBMDMs (*SI Appendix*, Fig. S3*E*), and this was impaired by *Tmem203* or *Sting* knockdown (Fig. 3*G*). However, IFN-I induction by DMXAA was only reduced by the knockdown of *Sting*, but not *Tmem203* (Fig. 3*G*), suggesting that *Tmem203* regulation of *Sting* may be ligand-dependent. To further test this, we used primary bone marrow-derived macrophages (BMDMs) isolated from WT or CRISPR-Cas9–targeted *Tmem203* knockout (KO) mice (*SI Appendix*, Fig. S3*F*) and stimulated them with DMXAA or 2′3′-cGAMP. In controls, both STING ligands induced a marked *Ifnb1* up-regulation (*SI Appendix*, Fig. S3*G*), but only 2′3′-cGAMP and not DMXAA mediated *Ifnb1* expression was reduced by *Tmem203* deficiency (Fig. 3*H*). In HeLa cells, which express lower levels of STING than the RAW 264.7 cells used in our initial screening (21), overexpression of TMEM203 alone did not elevate proinflammatory activities, as measured by the activation of a previously described (39) luciferase reporter, but it augmented overexpressed STING-induced responses (Fig. 3*I*). Constitutive overexpression of Tmem203 in RAW 264.7 cells (*SI Appendix*, Fig. S4*B*) significantly potentiated STING ligand-induced IFN activation (Fig. 3*J*). From the above data, we conclude that TMEM203 is a critical regulator of STING-induced type I IFN production and that its suppression impedes this process in response to specific STING ligands.

### TMEM203 Levels Regulate TBK1/IRF3 Activation Downstream of STING.

**Fig. 4.**
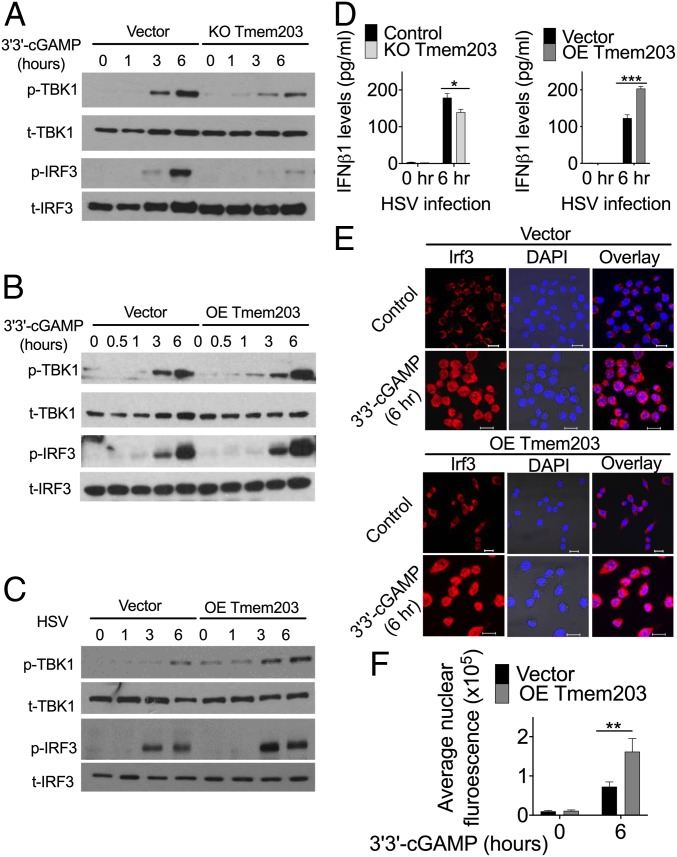
TMEM203 levels regulate TBK1/IRF3 activation downstream of STING. (*A*) CRISPR/Cas9-mediated Tmem203 knockout results in reduced TBK1 and IRF3 phosphorylation upon STING stimulation. Vector or CRISPR/Cas9-mediated Tmem203 knockout (KO Tmem203) RAW 264.7 cells were stimulated with 3′3′-cGAMP (1 µg/mL) for the time indicated. Activation of TBK1 and IRF3 was examined by Western blot analysis. Membranes were blotted with anti-phospho-TBK1 (p-TBK1), anti-total TBK1 (t-TBK1), anti-phospho-IRF3 (p-IRF3), and anti-total IRF3 (t-IRF3) as indicated (*n* = 3). (*B*) Increased TBK1-IRF3 activation in Tmem203-overexpressing RAW 264.7 cells in response to STING stimulation. Vector- or Tmem203-overexpressing (OE Tmem203) RAW 264.7 cells were stimulated with 3′3′-cGAMP (1 µg/mL) for the time indicated. Activation of TBK1 and IRF3 was examined by Western blot analysis. Membranes were blotted with anti-phospho-TBK1 (p-TBK1), anti-total TBK1 (t-TBK1), anti-phospho-IRF3 (p-IRF3), and anti-total IRF3 (t-IRF3) as indicated (*n* = 3). (*C*) Increased TBK1-IRF3 activation in Tmem203-overexpressing RAW 264.7 cells in response to HSV infection. Vector- or Tmem203-overexpressing RAW 264.7 cells were infected with HSV-1 for the time indicated. Activation of TBK1 and IRF3 was examined by Western blot analysis. Membranes were blotted with anti-phospho-TBK1 (p-TBK1), anti-total TBK1 (t-TBK1), anti-phospho-IRF3 (p-IRF3), and anti-total IRF3 (t-IRF3) as indicated (*n* = 3). (*D*) IFNβ cytokine secretion is impaired in Tmem203 knockout RAW 264.7 cells (*Left*) and is enhanced in Tmem203-overexpressing cells (*Right*) in response to HSV infection. CRISPR/Cas9-mediated Tmem203 knockout (or Vector) and Tmem203-overexpressing (or Vector) RAW 264.7 cells were infected with HSV-1 for 6 h, and Ifnb1 secretion was measured by ELISA. *n* = 3. (*E* and *F*) Tmem203 overexpression enhances IRF3 nuclear accumulation in macrophages in response to STING stimulation. Vector- and Tmem203-overexpressing RAW 264.7 cells were stimulated with 1 µg/mL 3′3′-cGAMP for 6 h. Subcellular localization of IRF3 was determined by IRF3 intracellular staining, and confocal fluorescence images were captured (*F*) (Scale bar: 10 µm). Average IRF3 nuclear fluorescence intensity of 50 to 200 cells was quantified using ImageJ (*F*). *n* = 3. *P < 0.05, **P < 0.01, ***P < 0.001.

To further characterize the functional contribution of TMEM203 to cGAMP-induced STING-signaling events, activation of TBK1 and IRF3 was compared between control and TMEM203-overexpressing or knockout RAW 264.7 cells. Each of these signaling molecules has previously been shown to be phosphorylated (and thus activated) in a STING-dependent manner, including responses to cytosolic dsDNA sensing (15, 40). While CRISPR/Cas9-mediated *Tmem203* knockout in RAW 264.7 cells (*SI Appendix*, Fig. S4*A*) resulted in an impaired TBK1/IRF3 phosphorylation (Fig. 4*A*), *Tmem203* overexpression (*SI Appendix*, Fig. S4*B*) augmented TBK1/IRF3 activation after 3′3′-cGAMP stimulation (Fig. 4*B*). Similar TMEM203-dependent changes were seen in these cells after human simplex virus-1 (HSV-1) infection (Fig. 4*C*), a dsDNA virus known to activate the STING/TBK1/IRF3 signaling axis (41). Consistently, HSV-1–stimulated IFNβ secretion was enhanced in *Tmem203* overexpressing cells (Fig. 4 *D*, *Right*) while it was impaired in Tmem203 knockout cells (Fig. 4 *D*, *Left*), confirming the functional significance of Tmem203 in the regulation of STING-IFN signaling.

Finally, we analyzed IRF3 activation downstream of STING by measuring the nuclear localization of IRF3 in control and *Tmem203*-overexpressing macrophages. Activated STING induces TBK1-IRF3 activation, leading to nuclear translocation of IRF3 that is critical for the initiation of transcription of the type I IFN genes (15). Elevated *Tmem203* expression in RAW 264.7 cells indeed led to an enhanced, 3′3′-cGAMP–induced IRF3 nuclear localization (Fig. 4 *E* and *F*), in line with the time frame observed for IRF phosphorylation (Fig. 4*B*).

### Transmembrane Domains of STING Are Required for the Formation of Its Complex with TMEM203.

**Fig. 5.**
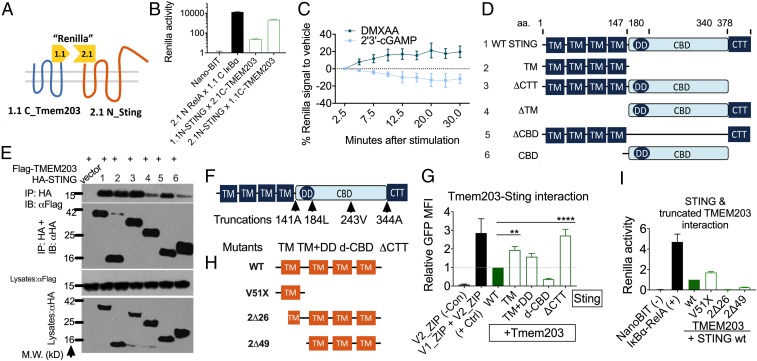
Molecular determinants of TMEM203-STING complex formation. (*A*) Detection of TMEM203-STING interaction by Renilla PCA. Tmem203 was tagged at its C terminus with the 1.1 (small) fragment of Renilla luciferase reporter while Sting was tagged at its N terminus with the 2.1 (large) Renilla fragment. (*B*) TMEM203 and STING interact in live cells. HEK293 T cells were cotransfected with the indicated fusion protein expression vectors; Renilla luciferase signal was detected by Nano-Glo live cell luciferase assay. Relative luminescence intensity was plotted compared with the negative control transfection of Nano-BIT construct. *n* = 3. (*C*) TMEM203-STING interaction is differentially regulated by DMXAA and 2′3′-cGAMP. TMEM203 and STING were tagged at their N termini with the 1.1 and 2.1 fragment of Renilla luciferase, respectively, to test for a molecular interaction between these proteins in live cells. Tmem203 and Sting were transfected into HEK293 T cells for 24 h and were then stimulated with DMXAA (50 µg/mL) and 2′3′-cGAMP (10 µg/mL) for the indicated time. Luciferase activity was calculated relative to Hoechst fluorescence (cell numbers) and calculated relative to the 2.5-min time point. *n* = 4. (*D* and *E*) TMEM203 coprecipitates with the STING N-terminal transmembrane region. WT and five mutant Sting constructs were created as indicated and fused with HA tags. CBD, CBD domain of Sting; ΔCBD, Sting without cyclic-dinucleotide binding domain (CBD); ΔCTT, Sting without cytoplasmic tail; ΔTM, Sting without transmembrane domain; TM, transmembrane domain. (*E*) HEK293 T cells were transfected with either empty vector, Flag-Tmem203, or HA-Sting (WT/mutants). Tmem203-containing complexes were immunoprecipitated (IP) using anti-Flag–coated beads and blotted for Flag and HA, as indicated. Lysates were also immunoblotted (IB) for Flag and HA. *n* = 2. (*F*) STING truncation mutants. Four serial truncation Sting mutants were individually created: after its N-terminal transmembrane domains (TM); after the dimerization domain (TM+DD); inside the cyclic dinucleotide binding domain (d-CBD); and before the C-terminal cytoplasmic terminal tail (ΔCTT). (*G*) TMEM203-STING association is not eliminated by C-terminal truncations of STING. HEK293 T cells were cotransfected with the Tmem203 and Sting WT/mutants cloned into the Venus vector system as described in Fig. 2*E*; Venus fluorescence signal was detected by flow cytometry. Relative mean fluorescence intensity was plotted compared with the STING WT–TMEM203 interaction. *n* = 4 to 7. (*H*) TMEM203 truncation mutants. Three mutants, expressing truncated versions of TMEM203 have been generated, as indicated. (*I*) AA1-51 of TMEM203 are sufficient to interact with STING. HEK293 T cells were cotransfected with the Tmem203 WT/mutants and Sting cloned into the Renilla reporter system as described in Fig. 5*A*. Renilla activity was measured 24 h posttransfection, and relative luciferase activity was calculated relative to Hoechst fluorescence (cell numbers). *n* = 3. **P < 0.01, ****P < 0.0001.

Having demonstrated the functional significance of TMEM203-mediated regulation of STING, we sought to further explore the mechanisms by which these two proteins interact and investigate the underlying mechanisms of selective, TMEM203-mediated regulation of STING activation. Since TMEM203 regulates cGAMP-, but not DMXAA-induced STING activation in macrophages, we proposed that these ligands may differentially influence the physical contact between TMEM203 and STING. We established the Renilla fragment protein complementation assay (Renilla PCA), which is based on expressing Tmem203 or Sting in fusion with either the small part (1.1) or the large part (2.1) of an engineered Renilla luciferase, NanoBit (Fig. 5*A*). The association of TMEM203 and STING causes a reversible assembly of a functional Renilla luciferase enzyme, which then catalyzes the breakdown of luciferin. A robust interaction between TMEM203 and STING was observed upon cotransfecting the Renilla PCA fusion constructs, and their strong association was demonstrated by comparison with the RelA–IκBα complex, which has been reported to form a stable complex in resting cells (Fig. 5*B*). Next, we coexpressed 2.1N-Sting and 1.1C-Tmem203 in HEK293T cells that were stimulated with either 2′3′-cGAMP or DMXAA. We detected a rapid, time-dependent reduction of the TMEM203–STING complex following cGAMP treatment, in contrast to an enhanced association of these proteins upon DMXAA treatment (Fig. 5*C*). This opposing effect of the two STING ligands on STING-TMEM203 association is likely to underlie the differential regulation of STING signaling by TMEM203 as demonstrated in BMDMs (Fig. 3 *G* and *H*).

TMEM203 is a 136-amino acid transmembrane protein with no obvious regulatory domains at the exposed short cytoplasmic regions. The protein sequence is highly conserved across vertebrates, with only a 3-amino acid difference in the sequence between the human and the mouse genes (22) (*SI Appendix*, Fig. S1). Thus, we speculated that the interaction between STING and TMEM203 is likely to be coordinated by STING, which contains complex regulatory domains. Although human and mouse STING are only 81% similar in primary sequence and 68% similar in amino acid composition (10), functional domains in STING are nonetheless spatially and structurally conserved across the two species. Previous studies on the structure of STING have identified the C-terminal cytoplasmic domain as the site for protein–protein interactions and ligand binding whereas the N-terminal transmembrane (TM) domains are mainly thought to be responsible for membrane anchorage (42). The cytoplasmic region of STING (approximately amino acids 153 to 378 in mouse) comprises of three domains: the dimerization domain (DD) (or helices α-5/α-6) formed by amino acids ∼155 to 180; the cyclic dinucleotide binding domain (CBD) formed by amino acids ∼153 to 340; and the cytoplasmic-terminal tail domain (CTT) formed by amino acids ∼340 to 378 (amino acids ∼340 to 379 in human) (4). The CTT is involved in TBK1/IRF3 binding and activation and is essential for type I IFN induction (15). To identify the molecular domains of STING required for its interaction with TMEM203, we created mutant Sting constructs that contain deletions of TM, CBD, or the CTT domain (Fig. 5*D*). Coimmunoprecipitation of HA-tagged WT or mutant STING with Flag-TMEM203 from HEK293 T lysates showed that the STING–TMEM203 complex is formed in the presence of STING’s TM domains (Fig. 5*E*, lanes 1 to 3 and 5) whereas the presence of CBD alone led to a very weak STING–TMEM203 interaction (Fig. 5*E*, lanes 4 and 6).

To substantiate these findings, 141A, 184L, 243V, and 344A Sting truncations were created (Fig. 5*F*), expressing TM domains only (TM), TM and dimerization domain (TM+DD), a disrupted cyclic dinucleotide binding domain (d-CBD), or CTT deleted STING (ΔCTT), respectively. Both the mutant and WT Sting were fused with the YFP expression plasmids, and their expression was tested by Western blot. Compared with the WT Sting, TM and ΔCTT mutants (*SI Appendix*, Fig. S5 *A* and *B*, lanes 3 and 6) showed enhanced protein expression whereas the TM+DD and d-CBD mutants showed reduced expression (*SI Appendix*, Fig. S5 *A* and *B*, lanes 4 and 5). Next, we fused the mutants and WT Sting with the V1 fragment of the previously described split YFP (Fig. 2*E*), which were cotransfected with the complementary V2-tagged Tmem203 expression plasmid into HEK293 T cells; the fluorescent signal was quantified by flow cytometry (Fig. 5*G*). To ensure that the overexpression of Tmem203 or Sting does not induce a nonspecific ER stress response (43), the level of spliced XBP1 gene, the presence of which is characteristic of a the unfolded protein response (44), was assessed in HEK293T cells, transfected with pcDNA3.1(control) or Tmem203-Sting PCA constructs. No substantial increase of XBP1 splicing was detected by RT-qPCR in Tmem203 and Sting expressing cells (*SI Appendix*, Fig. S6 *A*–*C*). Compared with the WT Tmem203–WT Sting interaction, a stronger protein interaction was observed between Tmem203 and Sting TM, TM+DD, and ΔCTT, respectively (Fig. 5*G*). However, d-CBD Sting showed an impaired interaction with Tmem203, due to reduced protein expression levels (*SI Appendix*, Fig. S5 *A* and *B*), potentially caused by degradation of dysfunctional STING protein. Of note, while the enhanced interaction between TMEM203 and STING-TM mutant was paralleled with high expression of this truncated STING protein, the TM+DD mutant exhibited similar association at a much lower STING protein expression level (Fig. 5 *F* and *G*).

To complement the above mapping of critical STING residues for the formation of TMEM203/STING complex, we generated mutants expressing truncated forms of TMEM203 (Fig. 5*H*). First, we verified that these mutants fused to EYFP are expressed at similar levels to the full-length TMEM203 when transiently transfected into HEK293T cells (*SI Appendix*, Fig. S5*C*). Next, we used the Renilla PCA assay to map the interaction between truncated TMEM203 mutants and STING. This analysis demonstrated that the N-terminal 51 aa of TMEM203 are sufficient to mediate the TMEM203/STING interaction (Fig. 5*I*).

In short, we conclude that interaction with TMEM203 is dependent on the N-terminal transmembrane domains of both STING and TMEM203 and that STING’s dimerization domain is potentially a significant regulator of their interaction.

### TMEM203 Localizes STING to Lysosomes.

**Fig. 6.**
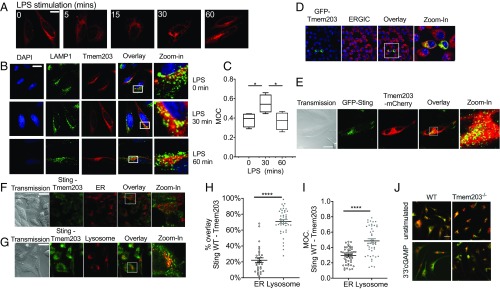
TMEM203 localizes STING to lysosomes. (*A*) LPS-induced perinuclear translocation of TMEM203. HeLa cells were transfected with TMEM203-mCherry fusion protein expression plasmid and stimulated with 1 µg/mL LPS for the stated length of time. (*B* and *C*). TMEM203 transiently colocalizes with LAMP1 in LPS-induced cells. HeLa cells were transfected with TMEM203-mCherry fusion protein expression plasmid and stimulated with 1 µg/mL LPS for the time indicated. LAMP1 localization was visualized using Alexa Fluor 488-conjugated anti-mouse secondary antibody. (Magnification: 63×.) Manders’ Overlap Coefficient (MOC) was calculated using ImageJ. Two-way ANOVA. (*D*) TMEM203 colocalizes with ERGIC. RAW 264.7 cells stably expressing GFP-TMEM203 were stained with anti-ERGIC-53/p58 (Sigma Aldrich), and confocal fluorescence images were captured. (*E*) TMEM203 colocalizes with STING. HeLa cells were cotransfected with Sting-GFP and mCherry-Tmem203 expression plasmids, and their localization was visualized by confocal microscopy. (*F*–*I*) Coexpressed STING–TMEM203 preferentially localizes to lysosomes. HeLa cells were cotransfected with the V1_Sting WT and V2_Tmem203 WT before ER (*F*) or lysosome (*G*) staining; fluorescence signal was detected with confocal microscopy at 80×. (*H*) Overlay of Tmem203-Sting signals and organelle signals were quantified with Fiji, and colocalization was calculated as the percentage of organelle with positive Tmem203-Sting detection. (*I*) Manders’ Overlap Coefficient (MOC) was calculated using ImageJ. Student’s *t* test. (*J*) STING fails to translocate from the ER in Tmem203 knockout BMDMs. BMDMs derived from WT or Tmem203 knockout C57BL/6 mice (*n* = 2 per genotype) were stimulated with 3′3′-cGAMP (10 µg/mL) for 3 h, and the overlap in STING localization (green) with ER (red) was visualized by confocal microscopy. Representative images are shown. *P < 0.05, ****P < 0.0001. (Scale bars: *A* and *B*, 10 μm; *E*–*G*, 20 μm.)

To further study the localization of TMEM203, HeLa cells were transiently transfected with mCherry-Tmem203. TMEM203 was predominantly found in perinuclear membrane structures in accordance with a previous report of ER localization (Fig. 6*A*) (22). Stimulation with LPS led to TMEM203 translocation to perinuclear, punctate membranes or vesicles (Fig. 6*A*). A more detailed analysis revealed a transient colocalization between TMEM203 and the lysosomal marker LAMP1 at 30 min stimulation with LPS in HeLa cells (Fig. 6 *B* and *C* and *SI Appendix*, Fig. S7). This rapid ER-to-vesicle translocation of TMEM203 correlates with the kinetics of translocation seen for STING during activation (45). When activated, STING has been shown to traffic to endo-lysosomal structures, termed Endoplasmic Reticulum Golgi Intermediate Compartments (46). Thus, we probed whether TMEM203 is expressed in this compartment and showed colocalization with the protein ERGIC-53 in RAW264.7 cells, stably expressing GFP-TMEM203 (Fig. 6*D*). Coexpression of GFP-STING and mCherry-TMEM203 also revealed a significant colocalization of these proteins (Fig. 6*E*). To characterize the localization of the TMEM203–STING complex, we cotransfected the previously described Venus PCA constructs of these two proteins (Fig. 2*E*) into HeLa cells and costained with either cell-permeable, lysosome- or ER-specific fluorescent dyes. The STING-TMEM203 complex was mainly expressed on lysosomal membranes rather than the ER (Fig. 6 *F*–*I* and *SI Appendix*, Fig. S8), a distribution consistent with the previously described localization pattern of activated STING (33). We therefore speculated that coexpression of TMEM203 and STING may preactivate or sensitize the STING pathway, thus leading to a lysosomal translocation, even in the absence of ligands. Finally, the impact of TMEM203 in cGAMP-induced STING trafficking was addressed in TMEM203^−/−^ BMDMs where we showed that STING is retained in the ER even after 3 h of 3′3′cGAMP stimulation (Fig. 6*J* and *SI Appendix*, Fig. S9). Thus, we concluded that TMEM203 expression is required for STING trafficking to lysosomes.

**Fig. 7.**
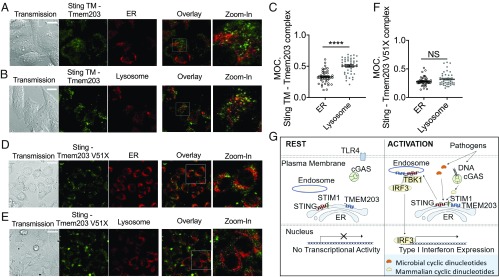
The C-terminal region (TM2-4) of TMEM203 is required for preferential localization of the STING–TMEM203 complex to lysosomes. (*A*–*C*) Localization of TM-STING/WT-TMEM203 complex. HeLa cells were cotransfected with the V1_TM-Sting and V2_WT-Tmem203 before ER (*A*) or lysosome (*B*) staining; fluorescence signal was detected under confocal microscopy at 80×. (*C*) Overlay of Tmem203-Sting signals and organelle signals was quantified with Fiji, and colocalization was calculated as the percentage of organelle with positive Tmem203-Sting detection. Manders’ Overlap Coefficient (MOC) was calculated as above. Student’s *t* test. (Scale bar: 20 µm.) (*D*–*F*) Localization of WT-STING/TMEM203-V51X. HeLa cells were cotransfected with the V1_WT-Sting and V2_V51X-Tmem203 before ER (*D*) or lysosome (*E*) staining; fluorescence signal was detected under confocal microscopy at 80×. (*F*) Overlay of Tmem203-Sting signals and organelle signals was quantified with Fiji, and colocalization was calculated as the percentage of organelle with positive Tmem203-Sting detection. Manders’ Overlap Coefficient (MOC) was calculated as above. Student’s *t* test. (Scale bar: 20 µm.) (*G*) Proposed molecular model for TMEM203 action. In the absence of immune stimuli, the type I IFN promoter has no transcriptional activity. Pathogen-released 3′3′-cGAMP or cGAS-produced 2′3′-cGAMP can induce the canonical STING pathway, resulting in TBK1-IRF3 interaction and subsequent type I IFN expression. Internalized LPS-bound TLR4 activates the “late signaling” events, which induce TMEM203-STIM1-STING interaction in the ER, leading to STING-TBK1-IRF3 activation and type I IFN expression. CDN, cyclic dinucleotide; cGAMP, cGMP-AMP; cGAS, cGMP-AMP synthase; ER, endoplasmic reticulum; IRF3, IFN regulatory factor 3; LPS, lipopolysaccharide; STIM1, Stromal Interaction Molecule 1; STING, Stimulator of IFN Genes; TBK1, TANK binding kinase; TLR4, Toll-like receptor 4; TMEM203, transmembrane protein 203. ****P < 0.0001; NS: not significant.

Using mutants expressing their C-terminal domains required for association in the YFP-PCA system, we tested the cellular distribution of the STING/TMEM203 complex. The TM-STING and wild-type TMEM203 complex showed a more equal localization between the ER and lysosomes (Fig. 7 *A*–*C* and *SI Appendix*, Fig. S10), compared with the WT-TMEM203/WT-STING complex (Fig. 6 *F*–*I*), likely due to the loss of STING’s regulatory site for posttranslational modification required to direct its trafficking. However, there was still a significant enrichment of this complex in the lysosomal compartment (Fig. 7*C*). In contrast, the complex between the N-terminal domain of TMEM203 (TMEM203 V51X) and WT-STING was no longer preferentially localized to lysosomes (Fig. 7 *D*–*F*), suggesting that residues 52 to 136 of TMEM203 are critical for driving the STING/TMEM203 complex to this compartment.

## Discussion

In this study, we show that an inflammatory regulator, TMEM203, forms part of a functional signaling complex with STING, thus regulating the activity of the effector kinase TBK1 and the transcription factor IRF3, leading to activation of type I IFN expression. TMEM203 acts independently of the canonical PRR systems, and the STING/TMEM203 complex localizes mainly in lysosomes. Our experiments aiming to further dissect the underlying molecular mechanisms in human and mouse macrophages revealed that TMEM203 regulates STING in a ligand-dependent manner. Upon STING activation by cyclic dinucleotides and HSV-1 but not by DMXAA, TMEM203 promotes the activation of TBK1 and IRF3. Finally, our data using truncated forms of STING and TMEM203 reveal that their N-terminal transmembrane domains are sufficient to form a complex and that this interaction is regulated by the α-helix/dimerization domain on STING. These experiments also confirm that the C-terminal region of TMEM203 is responsible for lysosomal trafficking of the TMEM203/STING complex. Supported by the mechanistic studies presented here, we propose a model (Fig. 7*G*) by which TMEM203-STING acts and promotes the TBK1-IRF3-IFN activation.

While detailed structural studies will be required to shed light on the exact molecular mechanisms by which TMEM203 regulates STING activity, our data demonstrate the important role for TMEM203 in ligand-dependent STING activation. In three primary and established macrophage systems, immunostimulation by cGAMP was impeded by the reduction/loss of TMEM203 while DMXAA-induced IFN-β expression only changed in parallel to Sting but not Tmem203 RNA levels. The mouse-specific ligand DMXAA binds to STING via mechanisms similar to cGAMP, but different amino acids are involved. Point mutations of hSTING S162A and E260I render it sensitive to DMXAA (47). Upon ligand binding, the STING dimer switches from an open-inactive to a closed-active conformation, the binding pocket being much tighter in the presence of 2′3′-cGAMP versus DMXAA (47). This model is further supported by our finding that DMXAA strengthens TMEM203-STING association while cGAMP weakens it.

Our data also highlight the role for TMEM203 in directing STING between intracellular compartments. While TMEM203 overexpression alone did not strongly enhance type I IFN expression in the mechanistic studies presented here, it has nevertheless promoted vesicular translocation of STING and its downstream IFN-β transcriptional activities. Recent literature suggests that the N terminus of STING is indispensable for its translocation from the ER to other membranes during stimulation (42). Thus, the association of this domain with TMEM203 may provide a mechanistic explanation for the importance of this STING domain. A recent study from Srikanth et al. reported that a calcium sensor STIM1 is critical for the retention of STING at the ER (19). Our finding that TMEM203 and STIM1 interact with each other and that they also compete for binding with STING suggests that these two proteins may together coordinate STING trafficking between intracellular membrane compartments, thus providing a critical control mechanism to prevent excessive IFN activation.

Host innate immunity is governed by a complex network of proteins cooperating in response to a variety of pathogens and autoimmune stimuli. Apart from the four well-established PRR systems controlled by TLRs, C-type lectin receptors, NOD-like receptors, and RIG-I–like receptors, innate immune signaling also utilizes a range of accessory/adaptor proteins to maintain the homeostasis of inflammation. STING is one of the major intracellular sensors of cytoplasmic double-stranded DNA and is a critical switch in the initiation of type I IFN production. A recent report suggested that cGAMP-induced STING activity enhances expression of inflammasome genes, thus providing a priming signal for activation of the NLRP3 inflammasome and the production of IL1β (48). However, RAW264.7 cells have been shown to harbor an inactivating mutation in the ASC1 adaptor protein that renders the caspase-1 inflammasome inactive in these cells (49). As TMEM203 is able to activate inflammatory signaling in RAW264.7 cells, we speculate that this protein is therefore unlikely to be involved in STING-mediated NLRP3 activation.

Recent work in mouse models of SLE has demonstrated that STING is required for homeostatic expression of negative regulators of immune activation (5) while analysis of human monocytes from SLE patients revealed a hyperactive STING signaling that is regulated by the IFN-induced gene, IFIT3 (24). Analysis of T cells (a critical source of type I IFN in this disease) (50, 51) from a cohort of SLE-positive, treatment-naive patients revealed unaltered STING and an elevated level of TMEM203, with a concurrent suppression of MAVS mRNA levels, suggesting that TMEM203 may be an important and previously unrecognized component in the pathology of SLE.

The physiological significance of STING has been demonstrated during infection with HSV, adenovirus (ADV), human papillomavirus (HPV), and negative-stranded RNA viruses, such as vesicular stomatitis virus (VSV) (2, 52). Gain-of-function STING variants lead to autoimmune, inflammatory diseases such as SAVI and FCL, manifesting clinically with symptoms of type I interferonopathy (12, 13). STING-mediated signaling is known to involve TBK1/IRF3 and NF-κB; targeting STING activity for therapeutic purposes nonetheless thus far focused on inhibiting downstream IFN receptors, and JAK/STAT (12, 13). Several cancer adjuvants showed promising antitumor effects in early clinical trials, but none has yet progressed to the clinic (53). Therefore, revealing TMEM203 as a STING-centered signaling regulator is of significant interest to both basic research to the understanding of diseases of IFN dysregulation and to the development of therapeutics and adjuvants modifying IFN induction.

## Materials and Methods

### Study Ethics.

All human tissue samples were collected and used under protocols approved by the University of Szeged Review Board (Ref. No. 2833/2011) and conformed to the declaration of Helsinki (1964 World Medical Association Declaration of Helsinki). Human blood was taken and used under protocols approved by the University of Sheffield Research Ethics Committee (UREC) (Ref. SMBRER310). All participants gave written informed consent.

Plasmid constructs were generated using standard techniques or described previously. For details, see *SI Appendix*. Primary cell isolation, culture, and manipulation followed standard protocols, as described in *SI Appendix*. Details of immunostaining and biochemical analyses are in the corresponding figure legends and in *SI Appendix*.

## Supplementary Material

Supplementary File
