# Supplementary material for: TMEM203 is a binding partner and regulator of STING-mediated inflammatory signaling in macrophages
Source: Proc Natl Acad Sci U S A. 2019 Jul 25;116(33):16479–88. doi: 10.1073/pnas.1901090116 (PMC6697806; doi:10.1073/pnas.1901090116)
Supplement: Supplementary File [file pnas.1901090116.sapp.pdf]

PNAS
www.pnas.org

Supplementary Information for

TMEM203 is a novel binding partner and regulator of STING mediated inflammatory signalling in macrophages

Yang Li, Sharmy J. James, David H. Wyllie, Claire Wynne, Agnes Czibula, Ahmed Bukhari, Katherine Pye, Seri Musfirah Bte Mustafah, Roberta Fajka-Boja, Eniko Szabo, Adrienn Angyal, Zoltan Hegedus, Laszlo Kovacs, Adrian V.S. Hill, Caroline A. Jefferies, Heather L. Wilson, Zhang Yongliang, Endre Kiss-Toth

corresponding authors: Zhang Yongliang and Endre Kiss-Toth
Email: miczy@nus.edu.sg; e.kiss-toth@sheffield.ac.uk

This PDF file includes:

Supplementary text
Figs. S1 to S10
Tables S1 to S2
References for SI reference citations

www.pnas.org/cgi/doi/10.1073/pnas.1901090116

Supplementary Information:

Methods:

Plasmids:

For details on previously described plasmid constructs used in these experiments, see (1). pGL4.*cxcl2* (1) and IL8-luc reporter vectors have been previously described (2). pGL4.EF1.rLuc was used as an internal control containing the human EF1 promoter from pEF-BOS (3). EYFP-PCA plasmid vectors have been reported previously (4). For Renilla-PCA, plasmids from the NonBIT-PPI (Promega) were used. Sequences of all vectors used were confirmed by sequencing.

SLE cohort

A cohort of patients diagnosed with SLE on the basis of the 1997 updated criteria of the American College of Rheumatology (5) were examined in this study and patient samples were followed-up at the Department of Rheumatology and Immunology, University of Szeged, Hungary. Patients aged 18-80 years with clinical disease activity in at least one organ system were eligible for the study. Exclusion criteria included the presence of an overlapping connective tissue disease, an infectious or other inflammatory process, and corticosteroid treatment with a prednisolone dose higher than 10 mg/day. Demographic characteristics and disease activity parameters of the cohort are presented in Supplementary Table 1. SLEDAI2K, anti-DNA, C3 and C4 complement levels in the patient serum were measured as part of the assessment of disease activity during lupus flare.

Isolation of primary macrophages

Monocytes were isolated from peripheral blood mononuclear cells (PBMCs) by positive selection using CD14-targeting magnetic microbeads (Miltenyi Biotec) and differentiated for seven days with 100 ng/ml human recombinant M-CSF (PeproTech). Bone marrow cells were cultured in the presence of 10% (v/v) L929 conditioned medium for 5 days to be differentiated into bone marrow-derived macrophages (BMDMs).

Isolation and analysis of T cells

PBMCs were isolated from SLE and healthy donors and stimulated with 1 µg/mL Phytohaemagglutinin (PHA-L, Sigma-Aldrich) for 72 hours. Total RNA was extracted from activated T cells and qRT-PCR was carried out using TaqMan Universal mastermix II (Applied Biosystems®) using product number: Hs01060665_g1 for ACTB, Hs01057884_s1 for TMEM203, Hs00736955_g1 for TMEM173, Hs00920075_m1 for MAVS.

Cell cultures, transient transfections

Human monocyte-derived macrophages (MDMs) were transfected with On-target Plus siRNAs (Dharmacon) against *TMEM203* using Viromer green transfection reagent (Lipocalyx). Immortalised bone marrow-derived macrophages (iBMDMs) were transfected with siRNA pool (Dharmacon) against mouse *Tmem203* or *Sting* as described for MDMs. RAW 264.7 cells (ATCC, TIB-71) were transfected using DharmaFECT transfection reagents (Dharmacon). HeLa cells were transfected with Lipofectamine 3000. HEK293 T cells were transfected using PolyFECT (Qiagen).

Generation of CRISPR/Cas9 Tmem203 Knockout RAW 264.7 cells

Four suitable sgRNA oligos were designed and used in the generation of pGL3-U6 sgRNA-Puromycin expression vectors. Transfection of RAW 264.7 with pGL3-U6 sgRNA expression vectors was carried out using Lipofectamine 2000. Colonies were selected for establishment of a stable cell lines along with CRISPR-Cas 9 control group using selective medium containing Puromycin (2 µg/ml) and Blasticidin (10 µg/ml).

Generation of Tmem203 Stably Expressing RAW 264.7 cells

Mouse *Tmem203* cDNA was cloned into the pcDNA 3.1 vector then transfected into RAW 264.7 cells using Lipofectamine 2000 (Invitrogen) and 500µg/ml G418 was used for the selection of stably expressing *Tmem203* cells.

STING ligand stimulation:

RAW 264.7 cells were transfected with 1µg 3'3'-cGAMP using Lipofectamine LTX (Invitrogen). For MDM, immortalised BMDM (iBMDM) and BMDM stimulation, cells were stimulated with 2'3'-cGAMP, 3'3'-cGAMP or DMXAA at the indicated doses and time. All ligands were purchased from Invivogen.

HSV-1 infection

RAW264.7 cells (control and Tmem203-overexpression / CRISPR-Tmem203 knockout) were infected with HSV-1 virus at MOI of 0.2.

Standard and quantitative real-time PCR

For analysis of mRNA levels following treatment, cells were washed with PBS and harvested for total RNA extraction using standard protocols. Complementary DNA was made from 500 ng total RNA (Biorad iScript cDNA synthetic kit) for gene expression analysis. Real-time quantitative PCR was performed using Primerdesign SYBR green mastermix in 10µl reaction volume in triplicate wells and quantified in BioRad

CFX384 Touch^{TM} real-time PCR cycler. Primers for amplification of human genes are listed in Supplementary Table 2.

Site-directed Mutagenesis and molecular cloning

Single-amino acid mutation was performed using standard protocols. Fidelity of the constructs was confirmed by Sanger sequencing. Primers used for mutagenesis are listed in Supplementary Table 2.

Protein complementary assay

To determine protein interaction using split Venus protein complementary assay (PCA), HEK293T cells were transfected. Post transfection, cells were harvested in FACS buffer and assessed by flow cytometry. Cell viability was determined by TO-PRO-3 (Thermo Fisher).

For split Renilla PCA, HEK293T cells were transfected. Post transfection, Renilla signal was analysed using Nano-Glo® Luciferase Assay System (Promega). Cells were stimulated with STING ligands for the indicated period and Renilla activities measured. For STIM1-TMEM203 competition with STING, increasing dose of pcDNA3.1-STIM1-his was transfected, as stated in the figure legend. Cells were also stained with Hoechst33324 (ThermoFisher) to indicate cell number for normalisation. Cells were maintained at 37℃ throughout the experiment.

Immunostaining and confocal microscopy:

HeLa cells were transiently transfected with Tmem203-mCherry for 48 hours using metafectene (Biontex) and treated with 1μg/ml LPS for 30 and 60 minutes as indicated. Cells were fixed and stained with an anti-LAMP1 antibody, (H5G11, SC-18821, Santa Cruz) and localisation was visualised using Alexa Fluor 488 conjugated anti-mouse secondary antibody (Molecular Probes). Bone-marrow isolated from WT and Tmem203$^{-/-}$ C57BL/6 mice were differentiated into BMDMs for 5 days using standard protocols and were then treated with 10 μg/ml 2'-3' cGAMP for 3 hours. Cells were fixed, permeabilised with 0.1% (v/v) Triton X-100 and stained with anti-STING antibody (Abcam ab181125) and ER or Lysosome Cytopainter staining kit (Abcam) as indicated. STING localisation in the ER or lysosomes was visualised using Alexa Fluor 647 conjugated anti-mouse secondary antibody (Abcam).

For live cell imaging of Tmem203-Sting organelle localisation, HeLa cells were transfected with V2_Tmem203 (WT or mutant) and V1_Sting (WT or mutant) for 18-24 hours using Lipofectamine 3000. Post transfection, samples were stained for 10 mins with ER or lysosome Cytopainter staining kit (Abcam). Images were acquired by confocal microscopy on a Zeiss LSM 510 META with 40X inverted water-lens (Molecular Probes). Images were analysed using Fiji software. ROIs were selected around the organelles to calculate the number of pixels of the organelle, Sting-Tmem203 fluorescence and co-localised signals. The percentage

of localisation was calculated as the ratio of co-localised pixels to the number of pixels occupied by the organelle.

RAW 264.7 macrophages stably expressing Tmem203 or empty vector were grown on coverslips and transfected with 1 µg 3'3'-cGAMP for 6h, along with controls. Cells were fixed with 3.7% (v/v) formaldehyde for 15 minutes at 37°C. Fixed and permeabilised cells were incubated with rabbit anti-IRF3 antibody (Cell Signaling Technology) before incubation with Alexa Fluor 546-conjugated donkey anti-rabbit IgG. Confocal fluorescence images were captured on a Zeiss LSM510 META microscope.

Human MDMs were permeabilised with 0.1% (v/v) Triton X-100 then washed and blocked followed by incubation with mouse anti- human CD68 (Dako) then with AlexaFluor 488-conjugated goat anti-mouse secondary antibodies. Nuclei were visualised with DAPI. Cells were imaged on a LEICA AFI6000 Time-Lapse microscope at 10X magnification.

Western-blotting and immunoprecipitation analysis

Post treatment, cells were harvested in RIPA buffer (Sigma Aldrich) and phosphatase inhibitors (Roche) and analysed using standard western blotting protocols.

For detection of target proteins, the following antibodies were used: TBK1 (Cell Signalling #3013 and #5483), anti-pIRF3 (Ser396) (Cell Signalling Technology), YFP (Cusabio, CSB-MA000283); Flag (M2, F1804, Sigma); Myc (Ab18185, Abcam).

Statistical analysis

Data was analysed using GraphPad Prism (8.0) and is presented as mean ± SEM, unless stated otherwise. (*: $p<0.05$, **: $p<0.01$, ***: $p<0.001$, ****: $p< 0.0001$).

Supplementary Figures

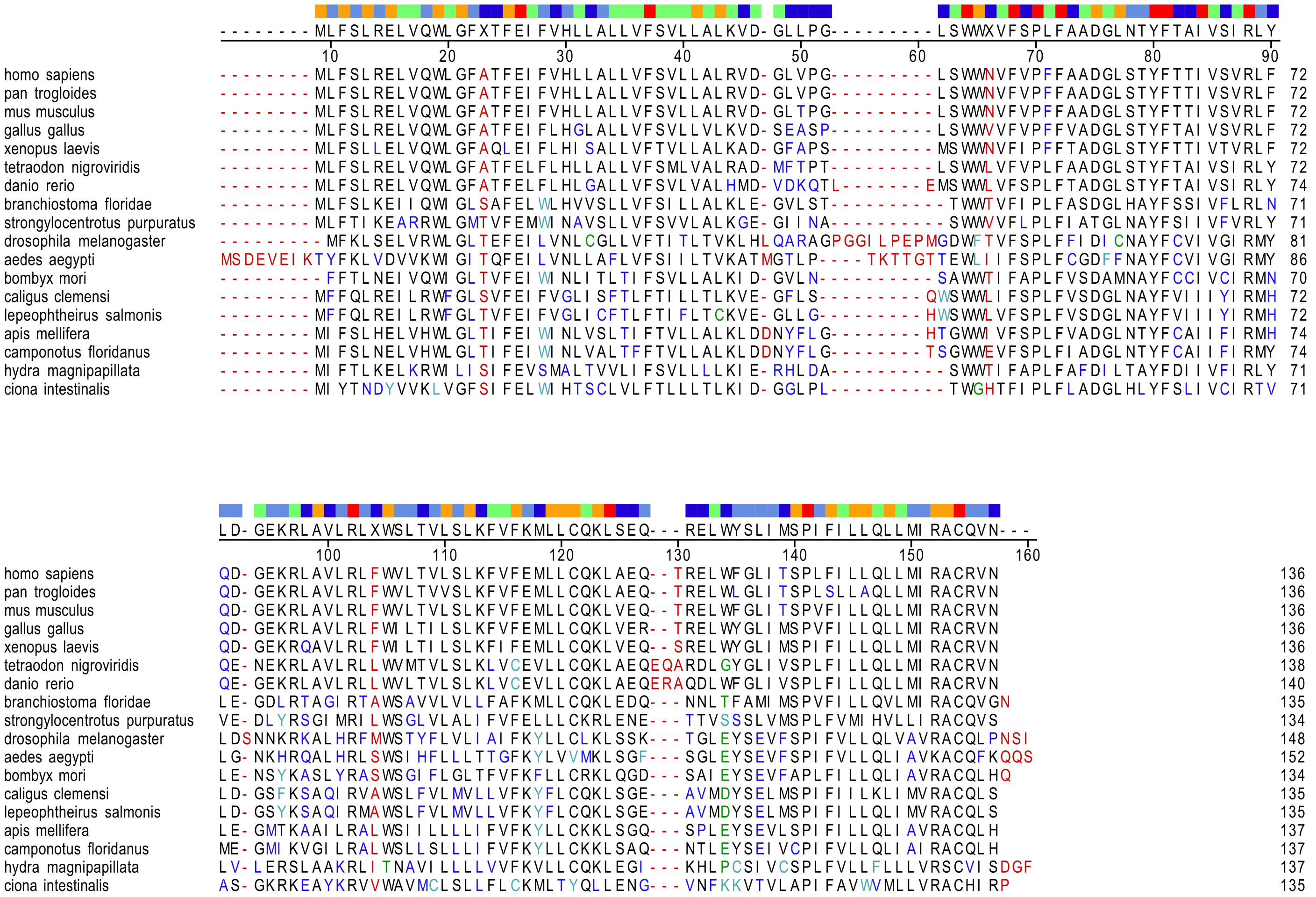

Fig. S1. **Alignment of TMEM203 orthologues:**

Homologues of the mouse TMEM203 protein were identified in GenBank and aligned using the Clustal W algorithm. Four transmembrane regions (TM1-4) were predicted by TMpred (6).

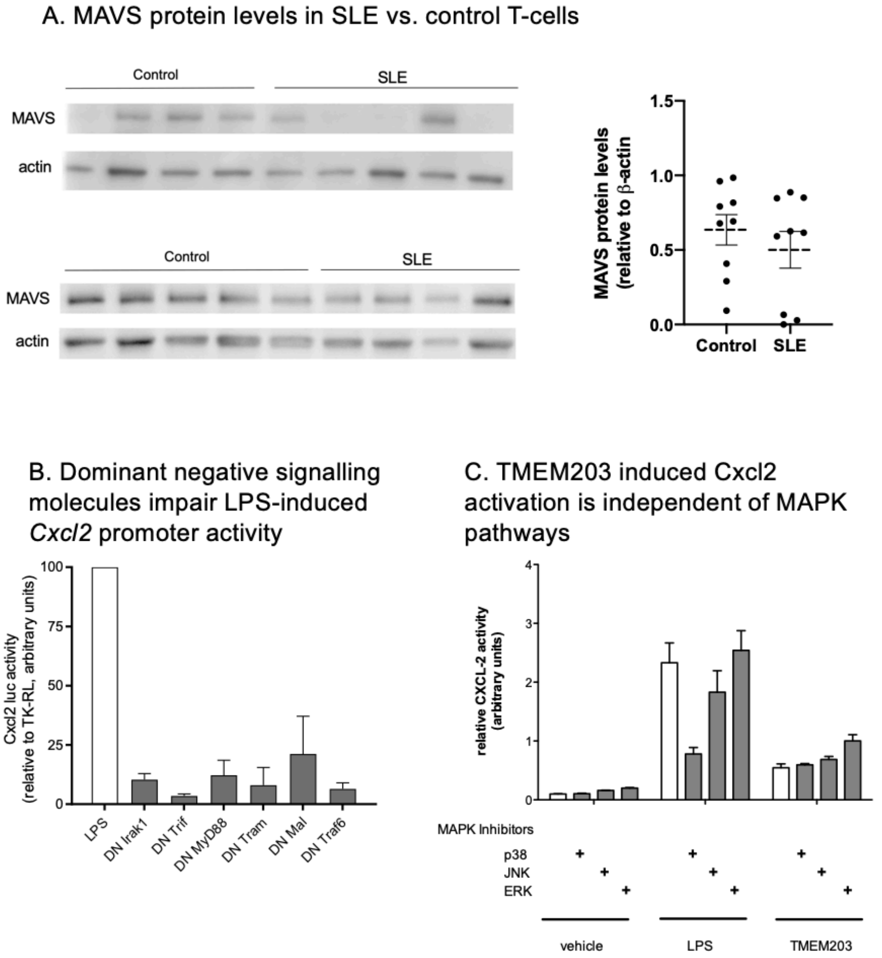

Fig. S2. MAVS protein levels in SLE and *Tmem203* induced *Cxcl2* activation

A. MAVS protein levels in activated T-cells. MAVS expression was analysed in PHA-L -activated T cells were assessed by western blotting from a pilot cohort of treatment-naïve systemic erythematous lupus (SLE) patients in comparison to cells from healthy individuals, using an anti-MAVS antibody (Santa Cruz, sc-166583). Each pair of lanes corresponds to a single patient; a total of nine controls and nine SLE patients' T cells were analysed. **B. Dominant negative signalling molecules impair LPS-induced *Cxcl2* promoter activity.** RAW 264.7 cells were transfected with the *Cxcl2*-pLuc and *EF1*-rLuc reporters, and the indicated expression plasmids encoding for dominant negative (DN) mutants of known pro-inflammatory molecules (mouse Irak1, Trif, MyD88, Tram, Mal and Traf6, respectively). LPS (100 ng/ml, 6 hours) was used as a positive control to test for the inhibitory activity of the DN constructs. Data is presented as mean ± SD, n=2.

C. Tmem203 induced Cxcl2 activation is independent of MAPK pathways. The impact of MAPK inhibition on Tmem203 overexpression vs. LPS induced cxcl2 expression was investigated in RAW 264.7 cells transfected with the cxcl2-pLuc and EF1-rLuc reporters. LPS (100 ng/ml) was added for 3 hours and used to induce cxcl2 luciferase reporter. Data is presented as mean ± SEM, n=3.

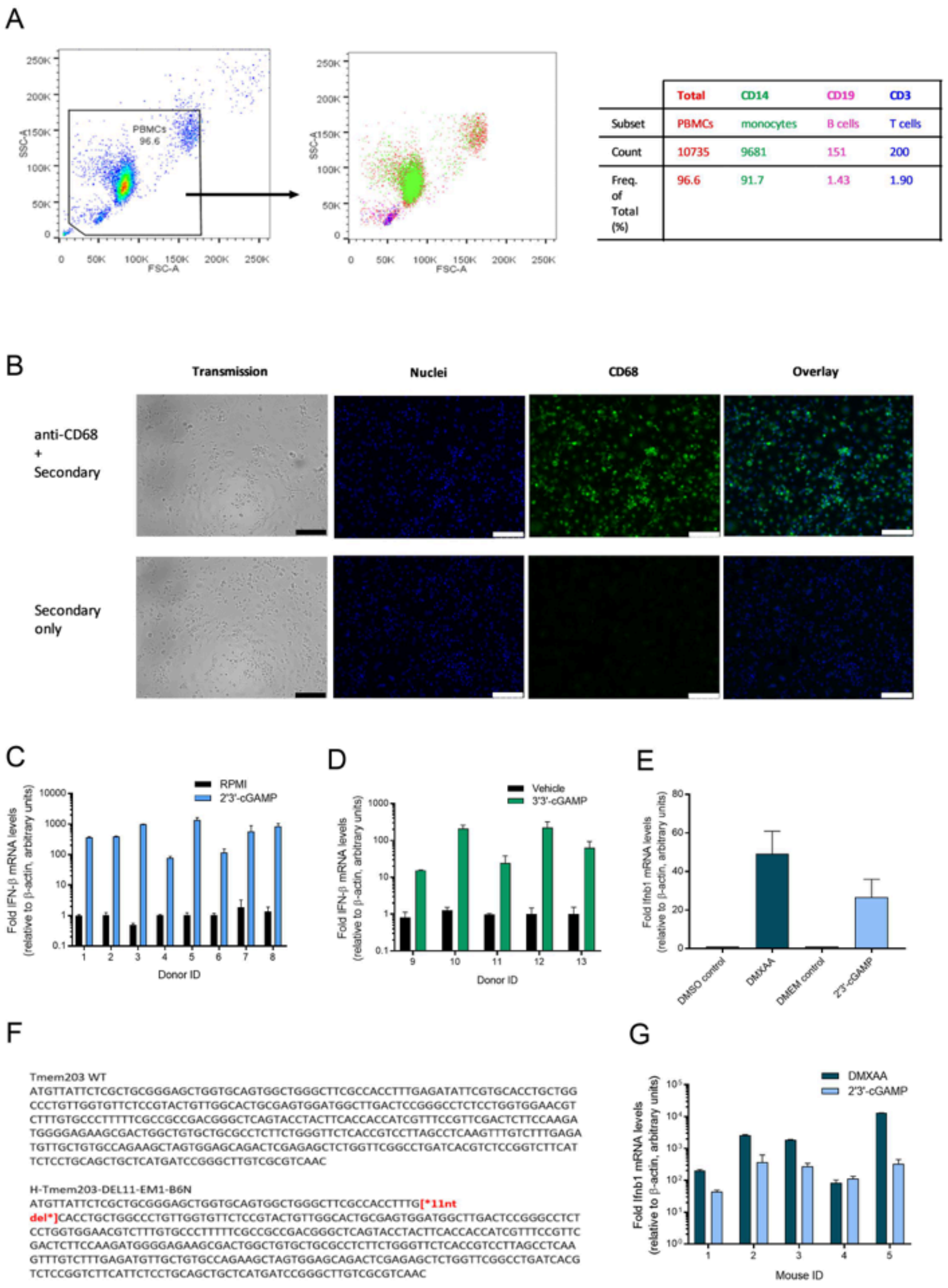

Fig. S3. Interferon response in human and mouse macrophages.

A. Assessment of high purity primary human monocyte isolated from whole PBMC. Post monocyte isolation, monocytes, B cells and T cells were stained with fluorescence-conjugated CD14, CD19 and CD3 antibodies, respectively. Over 90% purity of CD14 positive human monocytes were assessed by flow cytometry.

B. Human monocyte-derived macrophages express CD68. CD14 positive monocytes were differentiated into macrophages with M-CSF incubation and purity was examined by blotting with anti-human CD68 and detected with Alexa Fluor® 488 conjugated goat anti-human. Scale bar = 100 µm.

C. & D. TMEM203 knockdown reduces 2'3'-cGAMP (C) and 3'3'-cGAMP (D) induced IFN-β expression in MDMs. IFN-β production of control vs. stimulated MDMs was compared in individual blood donor MDMs. Mean IFN-β mRNA levels (relative to β-actin) ±SEM are plotted from 3 biological replicates for each MDM culture.

E. DMXAA (dark) and 2'3'-cGAMP (light) induces potent IFN-β mRNA increase in iBMDMs. IFN-β production of control vs. stimulated iBMDMs was compared. Mean Ifnb1 mRNA levels (relative to b-actin) ± SEM are plotted from n=3.

F. Genotype of *Tmem203* gene in the WT and *Tmem203* knockout mice. CRISPR-Cas9 induced deletion mutation of 11 nucleotides of exon ENSMUSE00000665152 (WT *Tmem203*) to generate a knockout allele (H-Tmem203-DEL11-EM1-B6N) of C57BL/6 background mice. Genotyping information confirmed by Sanger sequencing, MRC Harwell Institute, Oxford.

G. IFN-β response in BMDM stimulated with STING ligands. BMDM isolated from WT mice were stimulated with DMXAA (50 µg/ml) (dark) or 2'3'-cGAMP (10 µg/ml) (light) for 3 h. Mean IFN-β mRNA levels (relative to unstimulated controls) ± SEM are plotted from 3 biological replicates for each BMDM culture.

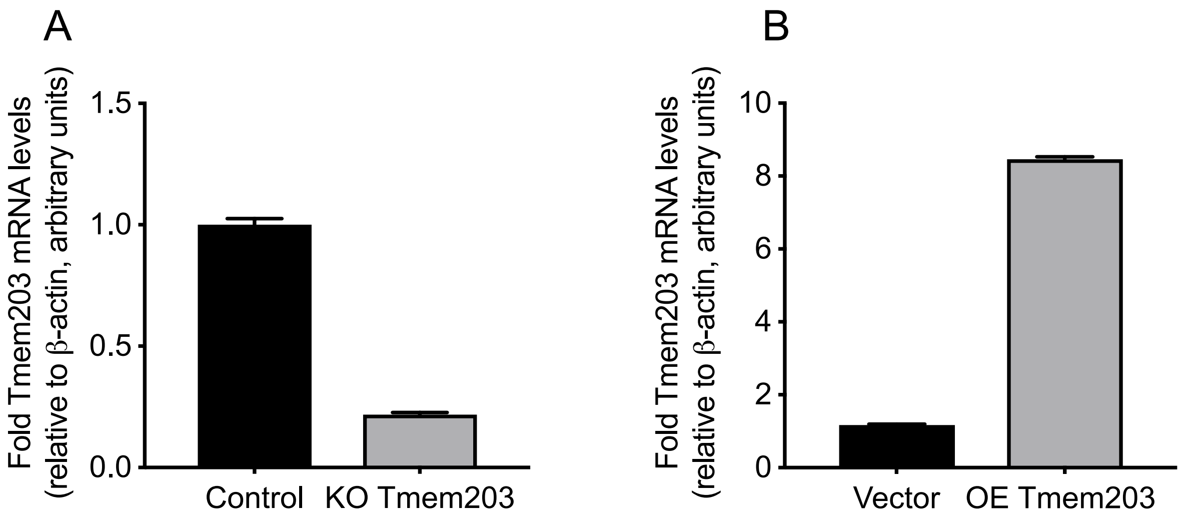

Fig. S4. Interferon response in human and mouse macrophages.

A. CRISPR/Cas9 TMEM203 Knockout RAW 264.7 cell line. CRISPR-Cas9 –directed Tmem203 knockout in RAW 264.7 cell line (KO Tmem203) showed a 3-4 -fold decrease in Tmem203 (against β-actin mRNA) expression determined by RT-qPCR and the cell line was established for further experiments.

B. Stable Tmem203 overexpression in RAW 264.7 cell line. Stable Tmem203 overexpression in RAW 264.7 cell line (OE Tmem203) showed a robust 8-fold increase in Tmem203 (against β-actin mRNA) expression determined by RT-qPCR and the cell line was established for further experiments.

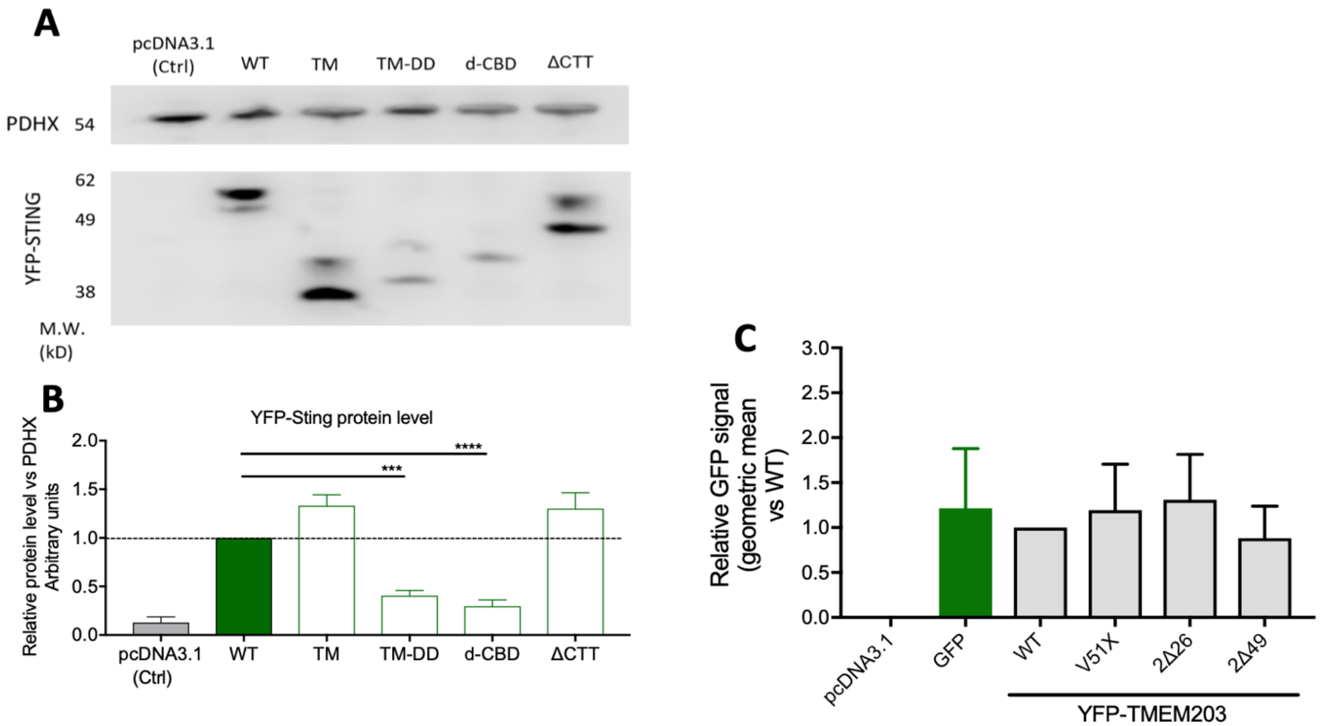

Fig. S5. Expression of truncated STING and TMEM203 mutants

A-B. C-terminal truncations alter STING protein expression. HEK293 T cells were transfected with the YFP fusion vectors expressing Sting WT and mutants as indicated. YFP-Sting protein levels were examined by western blot analysis. Membranes were blotted with anti-GFP and anti-PDHX (housekeeper) as indicated. Relative expression of mutant Sting was compared to WT expression. n=5.

C. Truncated TMEM203 mutants are expressed at consistent levels. HEK293 T cells were transfected with the YFP fusion vectors expressing TMEM203 WT and mutants as indicated. YFP-TMEM203 expression was examined by flow cytometry. Data is presented as mean ± SD, n=3.

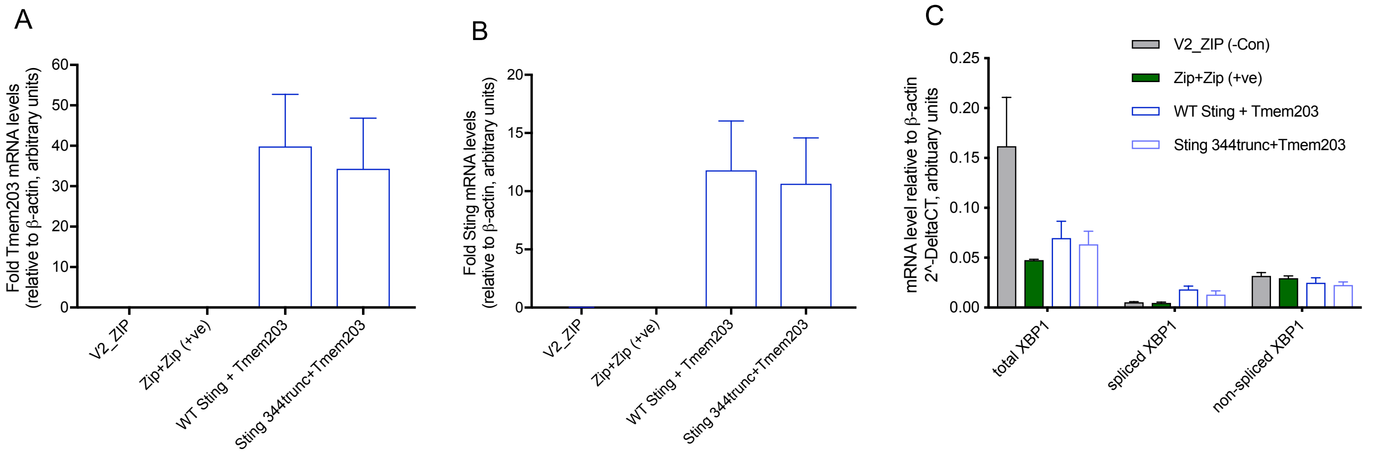

Fig. S6. Overexpression of Tmem203 and Sting does not induce ER stress.

S4. A-C. Venus PCA Tmem203 and Sting co-expression does not induce ER stress in HEK293 T cells. Co-transfection of Tmem203 (A) and Sting (B) Venus PCA constructs resulted in high expression of both genes but a relatively small increase of XBP1 mRNA splicing (C).

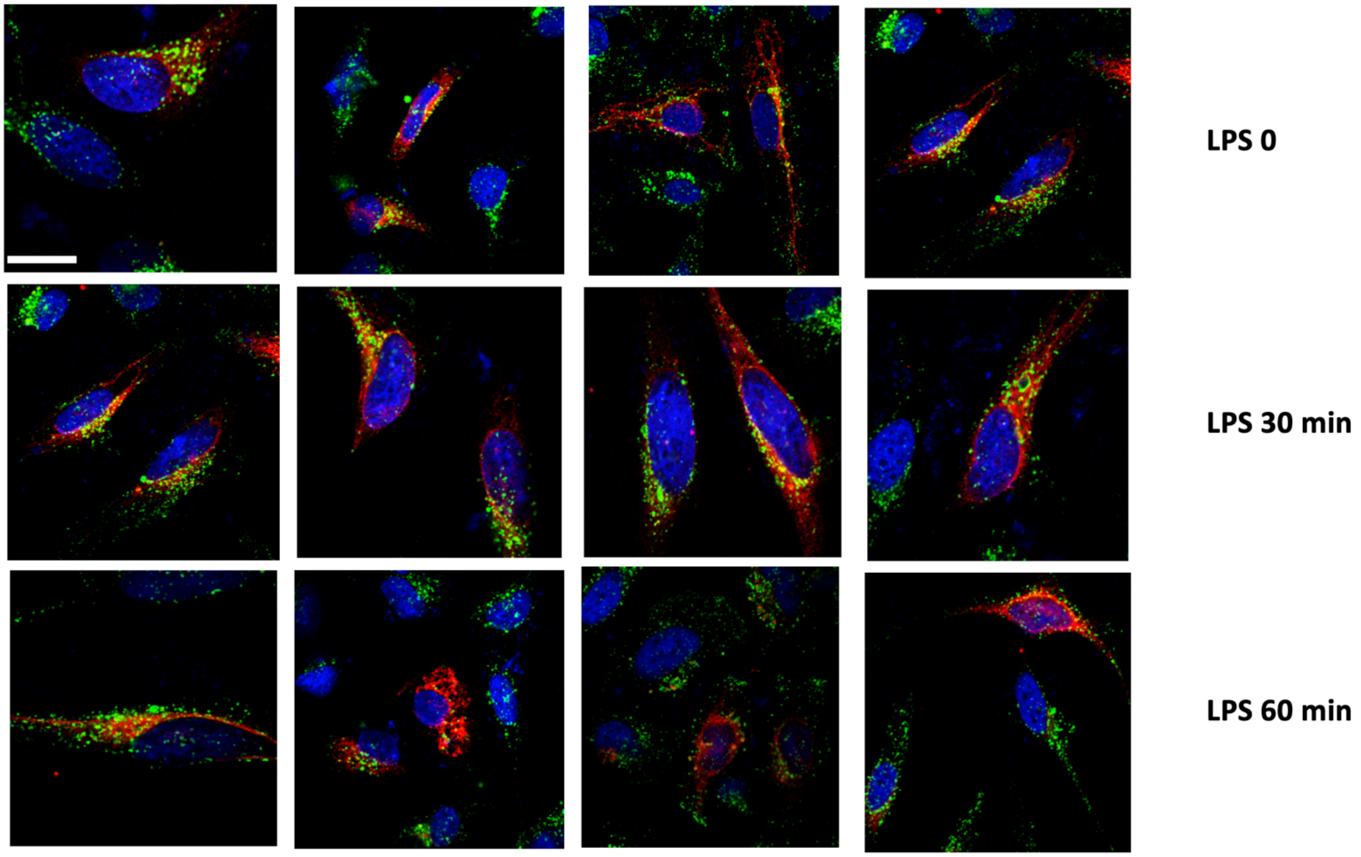

Fig. S7. TMEM203 transiently co-localises with LAMP1 in LPS induced cells.
HeLa cells were transfected with TMEM203-mCherry fusion protein expression plasmid and stimulated with 1 µg/ml LPS for the indicated time. LAMP1 localisation was visualised using Alexa Fluor® 488 conjugated anti-mouse secondary antibody. Images were taken under oil immersion at 63X magnification. Four images per condition (in separate columns) were used to quantify Manders' Overlap Coefficient. Scalebar: 10µm

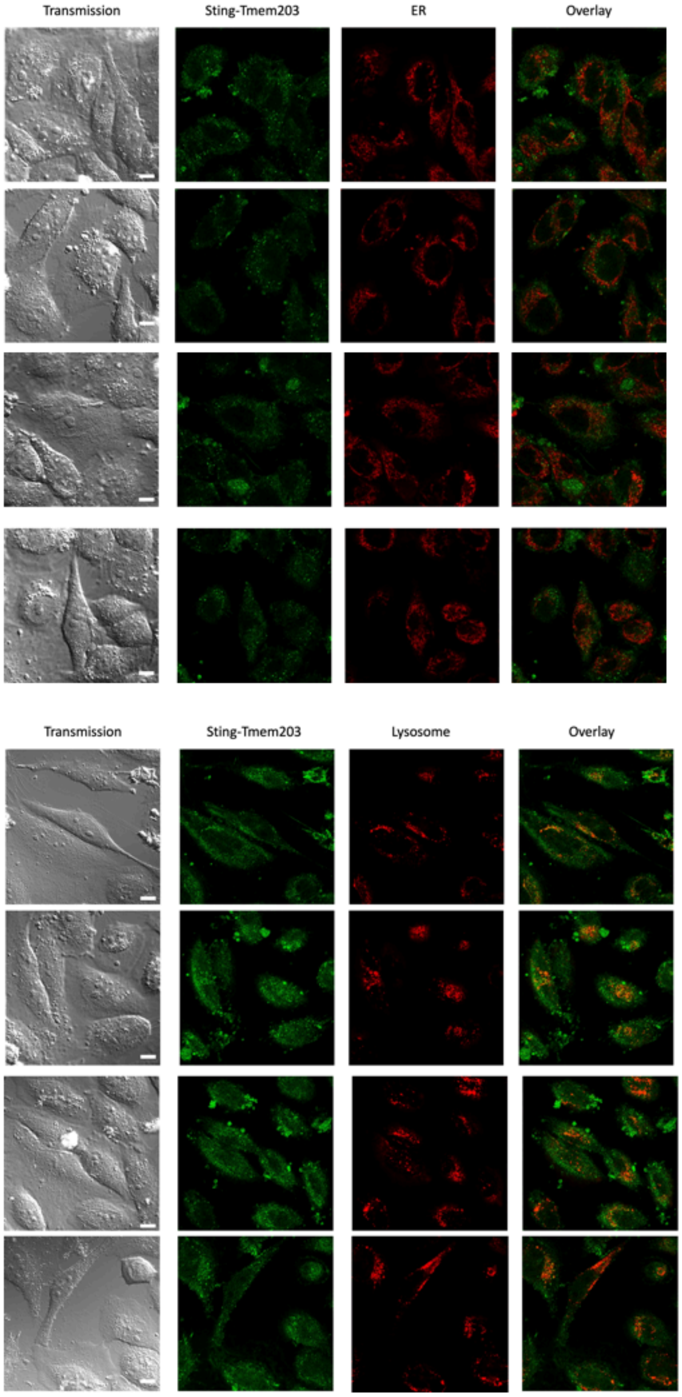

Fig. S8. Co-expressed STING–TMEM203 localises to lysosomes.

HeLa cells were co-transfected with the V1_Sting WT and V2_Tmem203 WT prior to ER or lysosome staining; fluorescence signal was detected under confocal microscopy at 80X. Images are representative of two independent experiments. Four images per condition (in separate rows) were used to quantify Manders' Overlap Coefficient. Scalebar: 10μm

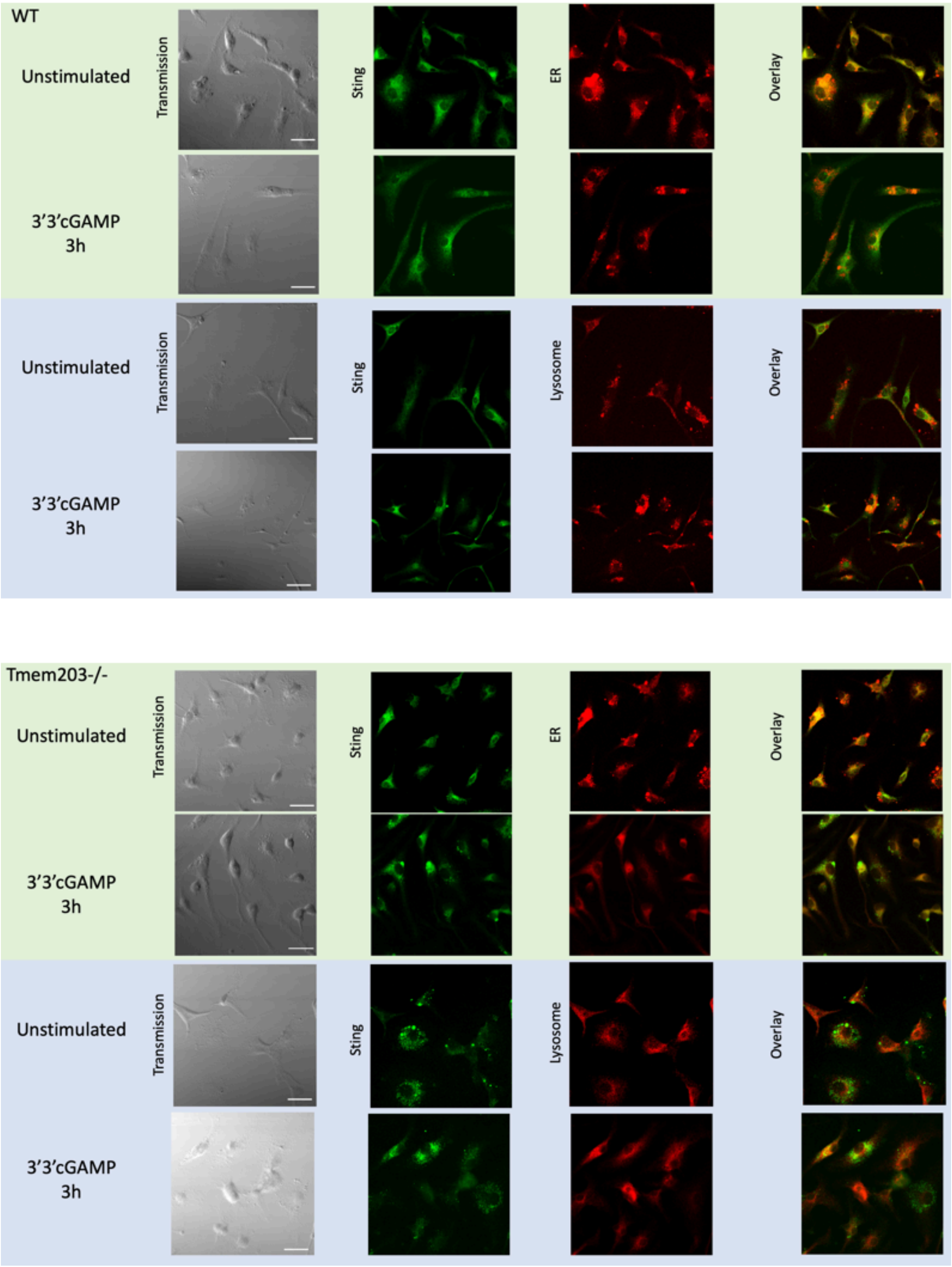

Fig. S9. STING fails to translocate from the ER in Tmem203 knockout BMDMs. BMDMs derived from WT or Tmem203 knockout C57BL/6 mice (n=2 / genotype) were stimulated with 3'3'-cGAMP (10 µg/ml) for 3 hours and the overlap in STING localisation with ER and lysosomes was visualised by confocal microscopy. Representative images are shown. Scalebar: 10µm

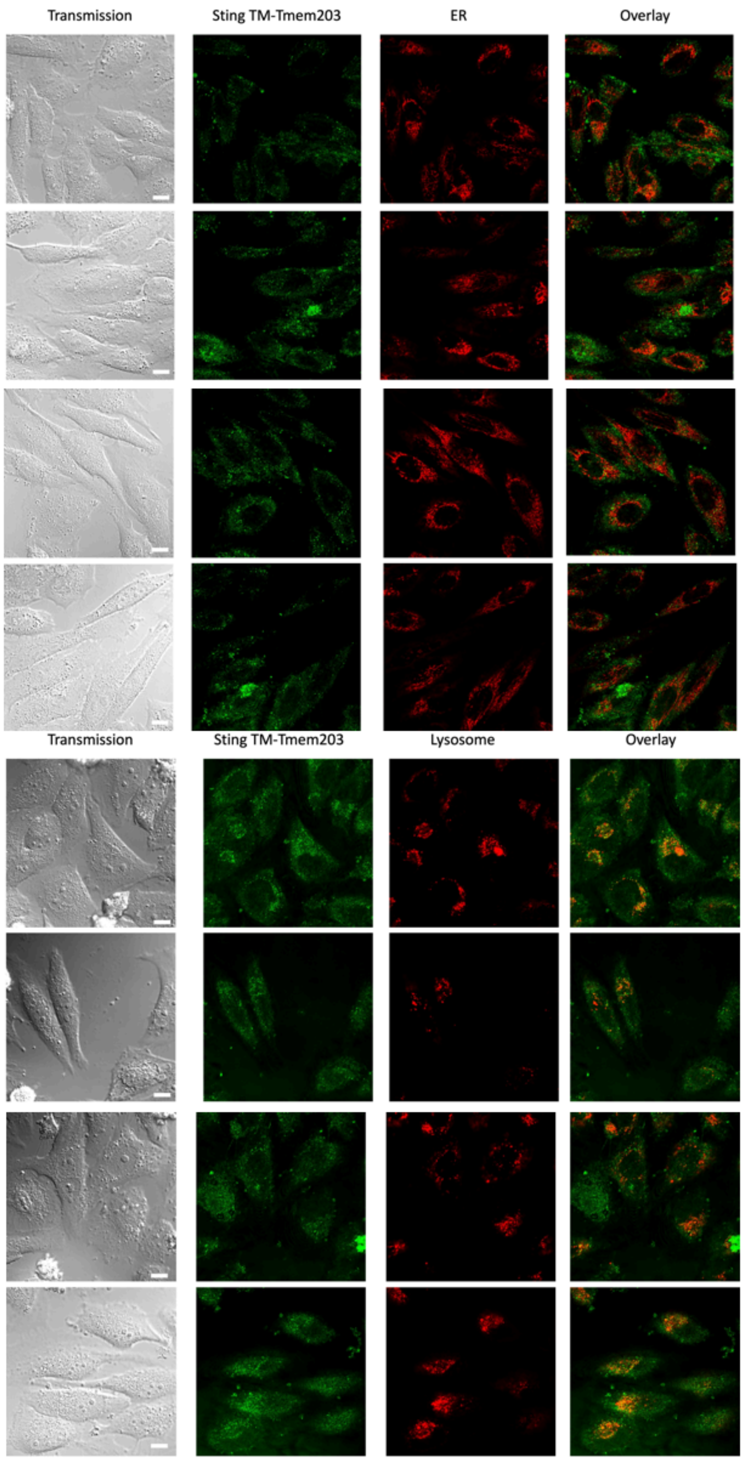

Fig. S10. Localisation of TM-STING/WT-TMEM203 complex.

HeLa cells were co-transfected with the V1_TM Sting and V2_WT Tmem203 prior to ER (A) or lysosome (B) staining; fluorescence signal was detected under confocal microscopy at 80X. Images are representative of two independent experiments. Four images per condition (in separate rows) were used to quantify Manders' Overlap Coefficient. Scalebar: 10μm

Table S1: Clinical description of SLE patient cohort. A cohort of 20 patients were diagnosed with SLE and clinical data were acquired. Asterisks denote data being presented as Mean±SD (Lower:Upper interquartile range). Abbreviation: F=female; M=male; SLEDAI-2K=systemic lupus erythematosus disease activity index 2000; Anti-DNA=serum level of antibody to double-stranded DNA; C3=serum C3 complement level; C4=serum C4 complement level; NA=not available.

Age at Sampling (Years)	Gender	SLEDAI-2K	Anti-DNA IU/ml	C3 mg/dL	C4 mg/dL
18	F	9	151	82	10
42	F	23	151	84	11
25	F	18	220	63	7
46	F	12	12	141	29
20	F	9	45	103	7
63	F	NA	74	131	11
37	M	16	220	80	15
36	F	16	220	59	15
35	F	NA	13	111	18
49	M	16	18	135	23
56	F	30	200	75	10
54	F	15	200	53	11
35	F	30	200	21	3
31	F	18	NA	NA	NA
45	F	8	14	41	10
59	F	2	10	90	20
37	F	19	220	67	12
52	F	17	200	63	15
38	F	6	41	116	23
30	F	NA	NA	NA	NA
N=20	F:18<br>M:2	*15.53±7.62<br>(9:18)	*122.72±87.63<br>(29.5:200)	*84.17±32.39<br>(63:107)	*13.89±6.41<br>(10:16.5)
			Normal range:<br>0-20	Normal range:<br>90-180	Normal range:<br>10-40

Table S2. List of primers

(A) RT-qPCR	
Genes	Primer sequences
Human IFN- β	F: 5’-AAGCAGCAATTTTCAGTGTCAGA–3’<br>R: 5’-CCTCAGGGATGTCAAAGTTCA–3’
Human IL-8	F: 5’- TGCCAAGGAGTGCTAAAG –3’<br>R: 5’- CTCCACAACCCTCTGCAC –3’
Human TMEM203	F: 5’- GTCTTCGAGATGCTGTTGTGC –3’<br>R: 5’- ACGTAATGAGGCCGAACCAG –3’
Human total XBP1	F: 5’- TGGCCGGGTCTGCTGAGTCCG –3’<br>R: 5’- ATCCATGGGGAGATGTTCTGG –3’
Human spliced XBP1	F: 5’- CTGAGTCCGAATCAGGTGCAG –3’<br>R: 5’- ATCCATGGGGAGATGTTCTGG –3’
Human non spliced XBP1	F: 5’- CAGCACTCAGACTACGTGCA –3’<br>R: 5’- ATCCATGGGGAGATGTTCTGG –3’
Human β-actin	F: 5’- GGATGACAGAAGGAGATCACTG –3’<br>R: 5’- CGATCCACACGGAGTACTTG –3’
Mouse Sting	F: 5’- GCTGGCATCAAGAATCGGGT –3’<br>R: 5’- TACTCCAGGATACAGACGCC –3’
Mouse Tmem203	F: 5’- CCCTGTTGGTGTTCTCCGTA –3’<br>R: 5’- GCACAAAGACGTTCCACCAG –3’
Mouse Ifnb1	F: 5’- TGTCCTCAACTGCTCTCCAC –3’<br>R: 5’- CATCCAGGCGTAGCTGTTGT –3’
Mouse Cxcl2	F: 5’- ATCCAGAGCTTGAGTGTGACG –3’<br>R: 5’- TTTGACCGCCCTTGAGAGTG –3’
Mouse β-actin	F: 5’- GGGACCTGACAGACTACCTCATG –3’<br>R: 5’- GTCACGCACGATTTCCCTCTCAGC –3’

(B) Site-directed Mutagenesis of Sting		
Gene	Mutation	Mutagenesis Primer Sequences
WT	WT<br>aa 1-379	F: 5’-AAAAGCTTATGCCATACTCCAACCTGCATCCAGCCATCCCACGGC-3’<br>R:5’AAACTCGAGTCAGATGAGGTCAGTGCGGAGTGGGAGAGGCTGA-3’
TM	TM1 – TM4<br>aa 1-146	F: 5’-AAAAAGCTTATGCCATACTCCAACCTGCATCCAGCCATCCCACGGC-3’<br>R: 5’-AAACTCGAGTCAGACTGCAGAGACTTCCGCTGG-3’
ΔCTT	TM1 – CBD<br>aa 1-338	F: 5’-AAAAAGCTTATGCCATACTCCAACCTGCATCCAGCCATCCCACGGC-3’<br>R: 5’-AAACTCGAGTCACTCCTTTTCTTCCTGACGAATGTGCC-3’
ΔTM	CBD – CTT<br>Aa 147-379	F: 5’-GGGAAGCTTATGTGTGAAGAAAAGAAGTTAAATGTTGCCCACGG-3’<br>R: 5’-AAACTCGAGTCAGATGAGGTCAGTGCGGAGTGGGAGAGGCTGA-3’
ΔCBD	Internal deletion of CBD<br>Aa 1-146, 340-379	F: 5’-AAGATATCGAGGTTACCATGAATGCCCCCATGACCTCAGTGGCA-3’<br>R: 5’-AAGATATCGACTGCAGAGACTTCCGCTGGAGTCAAGCTCTGAAGG-3’
CBD	CBD only<br>Aa 147-379	F: 5’-GGGAAGCTTATGTGTGAAGAAAAGAAGTTAAATGTTGCCCACGG-3’<br>R: 5’-AAACTCGAGTCACTCCTTTTCTTCCTGACGAATGTGCC-3’
141A	GCG->TGA<br>A -> STOP	F: 5’ CAGAGCTTGACTCCATGAGAAGTCTCTGCAGTC 3’<br>R: 5’ GACTGCAGAGACTTCTCATGGAGTCAAGCTCTG 3’
184L	CTA->TGA<br>L -> STOP	F: 5’ CGAATGTTCAATCAGTGACATAACAACATGCTC 3’<br>R: 5’ GAGCATGTTGTTATGTCACTGATTGAACATTCG 3’
243V	GTC->TGA<br>V -> STOP	F:5’ GTTTATTCCAACAGCTGATACGAGATTCTGGAG 3’<br>R: 5’ CTCCAGAATCTCGTATCAGCTGTTGGAATAAAC 3’
344A	GCC->TGA<br>A -> STOP	F: 5’ GAGGTTACCATGAATTGACCCATGACCTCAGTG 3’<br>R: 5’ CACTGAGGTCATGGGTCAATTCATGGTAACCTC 3’

(C) Site-directed Mutagenesis of Tmem203

V51X	aa 1-51V	F: 5’ GTGGAACGTCTTTTAGCCCTTTTTCGCCGC 3’<br>R: 5’ GCGGCGAAAAAGGGCTAAAAGACGTTCCAC 3’
L82X	aa 1-82	F: 5’ CGACTGGCTGTGTAGCGCCTCTTCTGGGTTC 3’<br>R: 5’ GAACCCAGAAGAGGCGCTACACAGCCAGTCG 3’
2D26	Tmem203 with aa 3-26 deletion	F: 5’ CCTTCACCATGTTATTGGTGTTCTCCGTA 3’<br>R: 5’ TACGGAGAACACCAATAACATGGTGAAGG 3’
2D49	Tmem203 with aa 3-49 deletion	F: 5’ CCTTCACCATGTTATTTGTGCCCTTTTTCG 3’<br>R: 5’ CGAAAAAGGGCACAAATAACATGGTGAAGG 3’

References to Supplementary Methods:

1. Larsen KC *et al.* (2009) Expression of tak1 and tram induces synergistic pro-inflammatory signalling and adjuvants DNA vaccines. *Vaccine* 27:5589–5598.
2. Wyllie DH *et al.* (2000) Evidence for an accessory protein function for Toll-like receptor 1 in anti-bacterial responses. *J Immunol* 165(12):7125–7132.
3. Mizushima S, Nagata S (1990) pEF-BOS, a powerful mammalian expression vector. *Nucleic Acids Res* 18(17):5322.
4. Eder K *et al.* (2008) Tribbles-2 is a novel regulator of inflammatory activation of monocytes. *Int Immunol* 20(12):1543–1550.
5. Hochberg MC (1997) Updating the American College of Rheumatology revised criteria for the classification of systemic lupus erythematosus. *Arthritis Rheum* 40(9):1725.
6. Ikeda M, Arai M, Okuno T, Shimizu T (2003) TMPDB: a database of experimentally-characterized transmembrane topologies. *Nucleic Acids Res* 31(1):406–409.
